# CAT HPPR: a critical appraisal tool to assess the quality of systematic, rapid, and scoping reviews investigating interventions in health promotion and prevention

**DOI:** 10.1186/s12874-022-01821-4

**Published:** 2022-12-26

**Authors:** Thomas L. Heise, Andreas Seidler, Maria Girbig, Alice Freiberg, Adrienne Alayli, Maria Fischer, Wolfgang Haß, Hajo Zeeb

**Affiliations:** 1 Leibniz Institute for Prevention Research and Epidemiology—BIPS, Bremen, Germany; 2 Health Sciences Bremen, University of Bremen, Bremen, Germany; 3 Institute and Policlinic of Occupational and Social Medicine, Faculty of Medicine, Technische Universität Dresden, Dresden, Germany; 4 Unit of Health Services Research, Clinic of General Pediatrics, Neonatology and Pediatric Cardiology, University Hospital Düsseldorf, Medical Faculty, Heinrich-Heine-University Düsseldorf, Düsseldorf, Germany; 5 Federal Centre for Health Education—BZgA, Cologne, Germany

**Keywords:** Critical appraisal tool, Evidence synthesis, Systematic review, Rapid review, Scoping review, Review of reviews, Mixed-methods, Meta-analysis, Health promotion, Prevention

## Abstract

**Background:**

For over three decades researchers have developed critical appraisal tools (CATs) for assessing the scientific quality of research overviews. Most established CATs for reviews in evidence-based medicine and evidence-based public health (EBPH) focus on systematic reviews (SRs) with studies on experimental interventions or exposure included. EBPH- and implementation-oriented organisations and decision-makers, however, often seek access to rapid reviews (RRs) or scoping reviews (ScRs) for rapid evidence synthesis and research field exploration. Until now, no CAT is available to assess the quality of SRs, RRs, and ScRs following a unified approach. We set out to develop such a CAT.

**Methods:**

The development process of the Critical Appraisal Tool for Health Promotion and Prevention Reviews (CAT HPPR) included six phases: (i) the definition of important review formats and complementary approaches, (ii) the identification of relevant CATs, (iii) prioritisation, selection and adaptation of quality criteria using a consensus approach, (iv) development of the rating system and bilingual guidance documents, (v) engaging with experts in the field for piloting/optimising the CAT, and (vi) approval of the final CAT. We used a pragmatic search approach to identify reporting guidelines/standards (*n* = 3; e.g. PRISMA, MECIR) as well as guidance documents (*n* = 17; e.g. for reviews with mixed-methods approach) to develop working definitions for SRs, RRs, ScRs, and other review types (esp. those defined by statistical methods or included data sources).

**Results:**

We successfully identified 14 relevant CATs, predominantly for SRs (e.g. AMSTAR 2), and extracted 46 items. Following consensual discussions 15 individual criteria were included in our CAT and tailored to the review types of interest. The CAT was piloted with 14 different reviews which were eligible to be included in a new German database looking at interventions in health promotion and prevention in different implementation settings.

**Conclusions:**

The newly developed CAT HPPR follows a unique uniformed approach to assess a set of heterogeneous reviews (e.g. reviews from problem identification to policy evaluations) to assist end-users needs. Feedback of external experts showed general feasibility and satisfaction with the tool. Future studies should further formally test the validity of CAT HPPR using larger sets of reviews.

**Supplementary Information:**

The online version contains supplementary material available at 10.1186/s12874-022-01821-4.

## Background

Reviews in health promotion and prevention research are primarily used to summarise, analyse, and assess various evidence sources for answering a particular research question and help to overcome the know-do gap in different implementation settings [1–3]. In particular, when there is a rapid increase in both primary research and other sources of information within a research field or when conflicting review results make conclusions less definitive, readers need assurance that the methods used in a review are designed to minimise bias [4]. Well-established review guidelines and standardised procedures can be used to increase both the quality and content of reviews [1, 2, 5–9]. Their uptake among researchers was profoundly influenced by the work of coordinated reporting guideline initiatives such as the EQUATOR network [10]. The PRISMA guideline, updated in 2020, is one of the most cited guidelines aiming to set minimal reporting standards. It provides guidance on how to transparently report review sections such as abstract, the search and synthesis methods to increase reproducibility, replicability and confidence in a review [8].

In addition to this development a separate line of research, first mentioned in the medical context more than three decades ago, established: the development and application of Critical Appraisal Tools (CATs), which is devoted to explore ways of assessing the quality of reviews [11–13]. Beyond being able to draw conclusions on the overall reporting quality of a review, the aim of using a CAT is to transparently and objectively assess the selection and application of adequate review methods and to identify major methodological shortcomings or bias, including the appropriateness of review conclusions. Covering methodological aspects of different review steps, this process can lead to an overall rating regarding the general quality of a review report [14, 15]. Various CATs have been developed over time for different fields of application and target audiences. They mainly differ in the degree of manualisation (i.e. guidance documents with further explanations to reach objective ratings), type of questions (e.g. open or closed), type of answers (including the number of answer options), usage of overall scores, and the effort and time required for completion [11, 13–39]. The area of application of CATs can be further classified into scientific use cases [15, 38], education purposes [20, 26] or guideline development [7, 30, 33]. We developed a new CAT for Health Promotion and Prevention Reviews (CAT HPPR) primarily to provide assessments for different review types found in a review database for health promotion and prevention. In the past, a similar approach has been taken by researchers of healthevidence.org, a curated review database for systematic reviews [14].

The “GKV-Bündnis für Gesundheit”, a joint initiative of all statutory health insurance funds for developing and implementing setting-based health promotion and prevention measures, commissioned a series of reviews on strategies and interventions for health promotion and disease prevention. Reviews focused on settings such as early child care, schools and the local community. Final reports were later compiled in a review database (“Knowledge for Healthy Settings”) and are available to the public (https://www.gkv-buendnis.de/forschung-im-buendnis/datenbank-wissen-fuer-gesunde-lebenswelten/). The commissioned reviews covered a broad range of topics and focused on different evidence sources (e.g. studies, case or best practice reports etc.) to be included. Led by decisions on content or scope specified by the “GKV-Bündnis für Gesundheit” to support health policy decision-making, the type of review and methods selected by the author teams were not all the same [40]. This mix in applied methods can also be seen in reviews published by international journals in the field of health promotion and prevention [41]. Existing CATs are predominantly designed to assess systematic reviews, and were not well suited to reflect on and evaluate unique key aspects of some emerging review types (e.g. scoping reviews) and complementary approaches (e.g. mixed-methods: integration of quantitative and qualitative data) which were part of this review collection [14, 15, 40]. More specifically, during our background search for published CATs, we also could not identify CATs which were exclusively designed to be used for assessing scoping reviews or rapid reviews. As a result, no standardised approach to critical appraise different review types existed at that time, which left end-users of reviews (i.e. users of curated review databases, guideline developers etc.) with imperfect solutions whenever a critical assessment for different review types was required. Either CATs for systematic reviews had to be used across different review types, where many criteria remained not assessable/applicable (e.g. appraisal of synthesis methods for Review of Reviews using AMSTAR) or a critical assessment of emerging review approaches was not undertaken — which left critical review evidence unassessed.

The lack of a well-documented CAT for simultaneous assessment of various reviews types motivated the development of this new tool. The goals of the project were: (i) to develop working definitions of review types in order to set the scope for the CAT HPPR, (ii) to develop an appraisal tool based on key criteria of existing CATs (e.g. healthevidence.org, AMSTAR 2) including a manual for end-users and (iii) to pilot the tool with a set of available reviews funded by the “GKV-Bündnis für Gesundheit”.

## Methods

The tool development process of CAT HPPR was pre-determined by a research protocol in German language (see Availability of data and materials). The reporting on the development process of CAT HPPR is informed by recommendations defined by Whiting et al. for developing quality assessment tools (“Stage 2: tool development”) [42]. Documentation of the search and selection process to identify and select CATs, reporting guidelines and items is appended to this article.

### Search for retrieving reporting guidelines and standards to define review types

For developing the CAT HPPR, we first used a pragmatic search in July 2019 of relevant websites (including the EQUATOR Network, Cochrane, JBI), an electronic database (Medline) and references provided by members of the larger project team in order to identify relevant reporting guidelines/standards (*n* = 3) [1, 2, 9]. We then collated further guidance documents for conducting a review within the scope of the tool (systematic reviews, rapid reviews, scoping reviews) and optional complementary review approaches (as review of reviews (also known as overview of reviews), with mixed-methods-approach, with meta-analysis) (*n* = 17) [5–7, 41, 43–55].

### Consensus approach to define review types

Based on identified documents, narrow working definitions for review types were developed and further refined, involving all project partners (tool developers, members of a project-specific reviewer pool piloting the novel CAT). Tool developers comprised all authors of this article (*n* = 8), whereas members of the reviewer pool (*n* = 3) were experienced review authors with methodological knowledge beyond systematic and Cochrane reviews recruited and commissioned by the “GKV-Bündnis für Gesundheit” (see Acknowledgements). Given the lack of consensus regarding the different types of reviews and their complementary approaches in the scientific literature, this step was crucial for achieving better applicability of the to-be-developed appraisal tool, its criteria and the global rating algorithm. Methodologically less narrowly defined types of reviews (e.g. overviews) or those that had a very large overlap with the types and approaches we had defined already (e.g. mapping reviews, umbrella reviews) were not considered separately [41].

### Search for retrieving CATs to inform items

As a further step towards identifying and tailoring relevant content of pre-existing CAT and their criteria for our tool, we carried out an electronic search using the same approach (i.e. Medline, websites, literature provided by project partners) as we did for reporting standards. Inclusion criteria for CATs were defined as follows: CAT originally developed for review articles, question/item-based CAT, CAT applicable to general medical or health topics, and CAT with corresponding guidance documents readily available.

### Compiling initial list of items for inclusion

We assessed 30 full-texts of CATs and other review evaluation instruments for eligibility. Excluded CATs were not exclusively developed to assess reviews (*n* = 1 [31];), mainly developed for training of practitioners (*n* = 3 [18, 19, 23];), developed for a certain medical field (*n* = 1 [25];), had no or limited guidance available (*n* = 3 [11, 21, 32];), were developed to assess the relevance of review findings (*n* = 2 [16, 26];), or were not considered for data extraction as the main report suggested strong overlap with another established CAT (*n* = 2 [22, 27];). As a result, 14 CATs [13–15, 20, 24, 28–30, 33–38] based on 18 reports were finally considered eligible for item identification. Included CATs were mainly developed for the quality assessment of systematic reviews. Since the CAT of healthevidence.org shared the most similar aim and content with our to-be-developed CAT [14], individual criteria of this tool were first extracted and compared to extracted criteria from the remaining 13 CATs. Extracted data was checked by a second tool developer. Criteria with the same wording or content across different CATs were removed.

### Initial items and scope

A review process of all criteria, including discussion among and consensus decisions by tool developers, led to a reduction in the number of identified individual criteria from 46 to 15. The following exclusion criteria informed the process of exclusion: strong overlap with items of healthevidence.org (*n* = 11; i.e. similar wording), limited relevance for quality of review findings (*n* = 16; e.g. “Were directions for future research proposed?” [36]), and limited potential for replicable assessments (*n* = 4; e.g. “Date of review – is it likely to be out of date?” [35]). The overall aim was to identify items which were comprehensive, relevant and objectively appraisable. Given some overlap between individual criteria in the set of extracted criteria, a factor analysis, as performed by developers of the original AMSTAR tool [34], was not undertaken. Instead, we extended some criteria with objectively appraisable content during further internal revisions of the tool (see Manual; coding boxes).

### First draft of CAT HPPR and guidance development

We also used reporting guidelines/standards as well as guidance documents for reviews for setting basic requirements for each criterion to be fulfilled by a review and developed further guidance for reaching a judgement by a user. A global rating system to combine information gained from all 15 criteria was introduced.

### Piloting and refinement

Finally, after piloting a first version of the CAT HPPR with 14 reviews, feedback and requests for further clarification by intended users of the tool’s assessment and experts of the project-specific reviewer pool led to final adjustments of the tool [40]. Feedback and requests were based on completed assessments among all major review types CAT HPPR was originally designed for (SR: *n* = 2, RR: *n* = 2, ScR: *n* = 10). As a result, a review-type specific algorithm was introduced in the global rating system in order to better take methodological advantages and disadvantages of individual review types into account. Among other things, the “Risk of Bias Assessment” was thus highlighted as a basic requirement and quality feature in systematic reviews compared to other review types. Informal feedback of CAT HPPR users was requested at the end of the piloting stage regarding processing time (not actually timed) and overall satisfaction with scope and applicability of CAT HPPR and its guidance documents.

## Results

### CAT HPPR

Table 1 provides all 15 questions (criteria) used in the novel CAT HPPR, whereas minimal requirements to obtain a positive rating are further defined in the manual and assessment form appended to this article.

**Table 1:** Criteria included in the CAT HPPR assessment form (critical criteria indicate those that, if not fulfilled, lead to substantial downgrading of a review)

	Criterion initially informed by	Critical criteria for SRs	Critical criteria for RRs, ScRs
C1. Is the review based on a clear and focused question that has been adequately formulated and reported?	healthevidence.org CAT	**X**	**X**
C2. Were methods of this review transparently reported prior to conduct of the review?	AMSTAR 2	**X**	**X**
C3. Were appropriate in- and exclusion criteria used in the selection process (title−/abstract and full text screening) of evidence sources (i.e. scientific work: studies, reviews, project reports, etc.)?	healthevidence.org CAT	**X**	**X**
C4. Was a search strategy for databases and/or other sources of information reported by the authors which can be considered as comprehensive?	healthevidence.org CAT	**X**	**X**
C5. Was the selection process of evidence sources from search to synthesis transparently reported?	AMSTAR 2	**X**	**X**
C6. Is a description of characteristics of included evidence sources provided in the review (especially PICO(−TSSD) or PCC elements)?	AMSTAR 2	**X**	**X**
C7. Were appropriate methods used to combine or compare the results of included evidence sources?	healthevidence.org CAT	**X**	**X**
C8. Do the results of the included evidence sources support the interpretation of the review authors?	healthevidence.org CAT	**X**	**X**
C9. Did the review team follow the four-eyes principle for important steps within the review process to reduce the risk of errors and biased decisions?	AMSTAR 2	**X**	
C10. Was a methodological quality assessment of included evidence sources, based on established criteria or a tool, part of the review?	healthevidence.org CAT	**X**	
C11. Was the risk of bias of included evidence sources incorporated in the presentation and discussion of the review findings or were strengths and weaknesses of the evidence sources critically discussed?	AMSTAR 2		
C12. Were homogeneity or heterogeneity of included evidence sources adequately considered in the review process and sufficiently presented in the final review?	healthevidence.org CAT		
C13. Were methodological limitations of the selected review type and methods sufficiently addressed in the discussion?	AQASR		
C14. Were potential conflicts of interest (including funding) of the review authors provided in the review or actively declared as non-existing?	AMSTAR 2		
C15. Were all relevant outcomes, including negative/adverse aspects of the object of consideration, mentioned?	SURE		

The new CAT HPPR was primarily influenced by the healthevidence.org CAT (i.e. items, plain language style) [14] and AMSTAR 2 (i.e. global rating process based on critical criteria) [15], which were originally designed to be used with systematic reviews exclusively. A major challenge remained to adapt the basic concept behind seven of the original healthevidence.org CAT items [14], six unique items of AMSTAR 2 [15], one item of AQASR [36] and one item of SURE [35] to be also applicable to other review types, namely rapid and scoping reviews, and complementary review approaches (as review of reviews, with mixed-methods-approach, with meta-analysis). Definitions for review types and complementary review approaches applicable to CAT HPPR can be found in appendix 1 of the tool’s manual. We used working definitions based on reporting guidelines and review specific methodological research to further narrow down minimal requirements for reaching a judgement.

A full rationale for inclusion of each of the 15 criteria is provided in the manual. To briefly summarise, C1 aims to assess whether review authors were able to provide an adequately formulated review question [8]. In addition to PICO(−TSSD), scoping reviews can be also assessed based on the PCC (population, concept and context) question format [5]. The gold-standard of whether the review was based on a protocol which details in advance the review’s rationale, objective and methods is subject of C2 [8, 56]. A review should have clearly reported in its methods section eligibility criteria by which the included evidence sources were selected and non-relevant sources were excluded to make results plausible and reproduceable (C3) [8, 9]. A well-documented and comprehensive search strategy includes search approaches for multiple literature databases and other search streams to identify relevant evidence sources, this can particular differ between systematic and rapid reviews which can be assessed with C4 [57]. Full reporting on the selection process is subject of C5 [8]. The description of characteristics of included evidence sources can be part of the results (e.g. in scoping reviews) as well as an intermediate step informing the synthesis of a review [5, 8]. C6 asks whether these characteristics are sufficiently reported in a review. Depending on the included evidence sources and the research question of interest, different approaches are available for synthesising the results (or data) in a review. Review authors should at least report a well-balanced narrative synthesis considering all included evidence sources (presented in tables and/or text) for a positive rating of C7 [5]. C8 investigates whether the interpretation by the review authors was in line with data of the included evidence sources. Involvement of at least two people in relevant review tasks (e.g. selection process) can help to avoid errors, biased decisions and, thereby, contribute to the overall decision quality which is assessed for a review at C9 [6]. Assessing potential bias of the included evidence by conducting a quality assessment can be considered as a review result on its own and helps to further understand the certainty of the evidence [6]; minimal requirements towards a quality assessment are outlined at C10. Strengths and weaknesses of the synthesised evidence can be based on this quality assessment and should be identifiable in the interpretation of the review findings (C11). Assessments on whether statistical tests and/or narrative reporting on homogeneity or heterogeneity of included evidence sources (which can guide selection of a synthesis method and clarify whether in- and exclusion criteria were thoroughly followed) were conducted are subject of C12 [15]. A critical reflection regarding limitations and decisions made in the review process is necessary to assess the uncertainty of the overall review results and to identify aspects of what future research should investigate. Limitations may stem from external factors (e.g. funding of the review project) and the context of the research, but, nevertheless, should be transparently reported (C13) [8]. Conflicts of interest affecting a review team (authors) can sometimes, not automatically, lead to biased decisions in conduct of a review, which can ultimately translate into biased review results and should be checked at C14 [15]. Finally, review authors should remain as objective as possible whilst taking both positive and negative aspects as well as unintended or adverse effects of interventions or exposures into account [46]. An unbalanced or no representation in the reporting of all relevant outcomes will lead to a negative rating of C15.

### Using the CAT HPPR

**Fig. 1:**
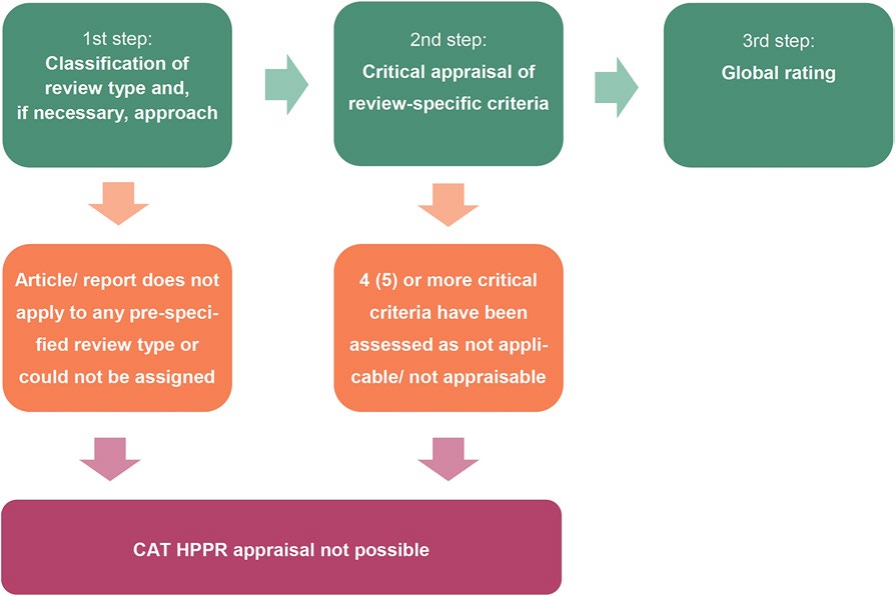
Critical appraisal process using CAT HPPR

Each assessment starts with an assignment for or definition of the review type (and, if applicable, the complementary review approach based on this type) which forms the basis for the entire appraisal process to come (see Manual). This is a basic requirement so that each criterion and question can be rated or answered in accordance to the methodological requirements for a particular review type and, if applicable, approach (Fig. 1).

**Fig. 2:**
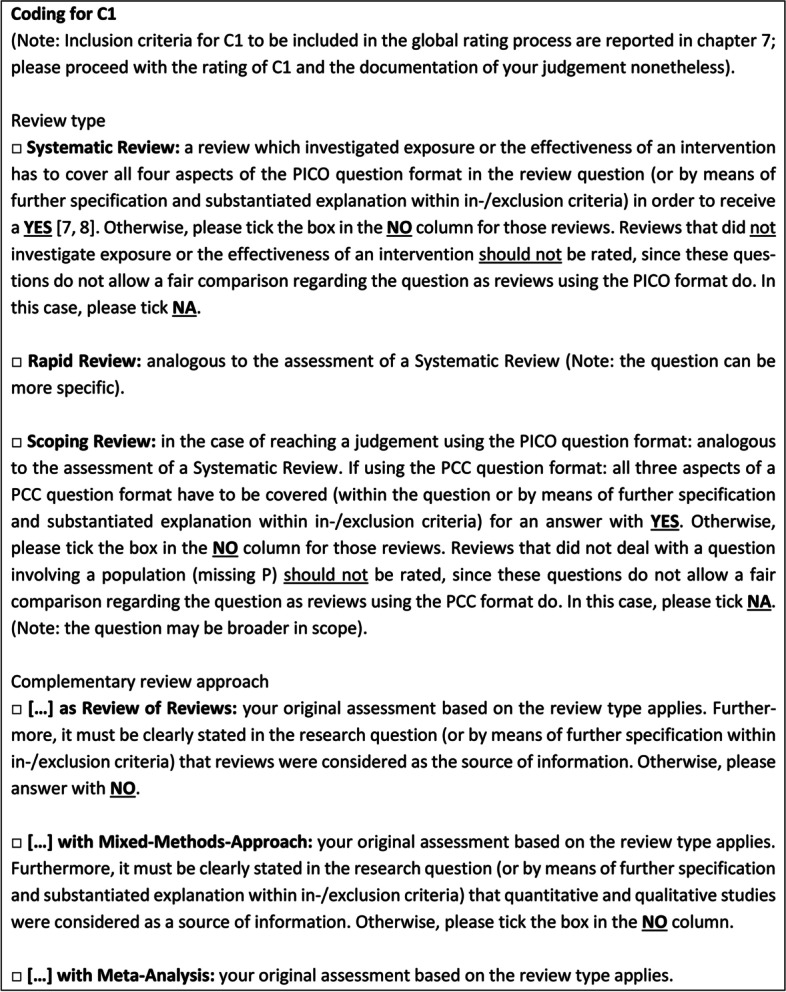
Example of a “Coding box” for C1

The CAT HPPR dictionary of the manual provides guidance on how to reach a judgement for a specific criterion. The information in the text box in particular serves as a guideline for reaching the final judgement. In addition to the obligatory consideration of “hard” criteria (i.e. information for reaching a judgement of YES or NO), further information, provided in the introduction to each criterion, serves as general orientation as to which factors may also influence the rating [14] (Fig. 2).

### Differences in ratings for systematic reviews, scoping reviews and rapid reviews

**Fig. 3:**
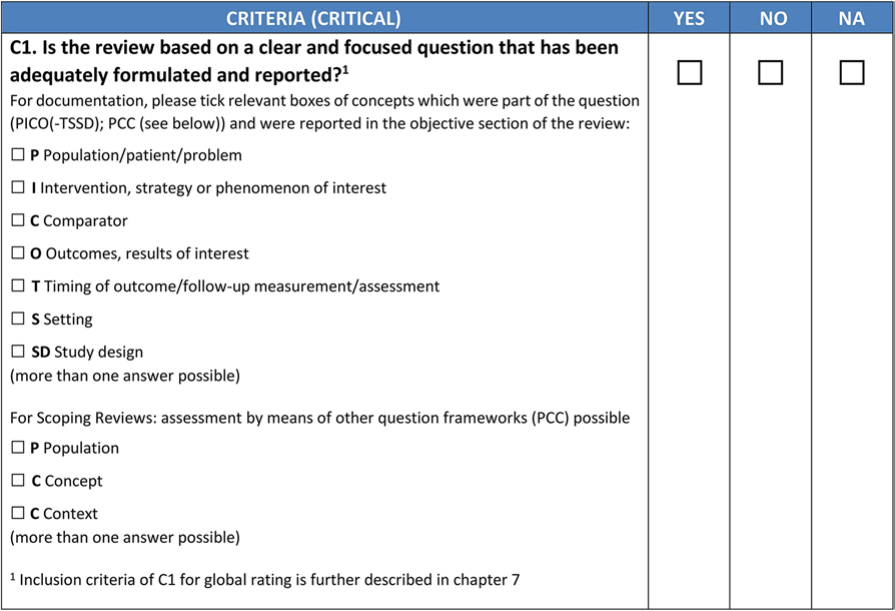
Example of the assessment form for C1

Not only do minimum requirements for individual criteria in CAT HPPR differ by design across assessments for different review types, but also the global rating is affected (Fig. 3).

Rapid reviews usually feature a reduced scope of the research question, a reduction in number of databases searched and use of search limiters (e.g. time period covered, language restrictions), the omission of the four-eyes principle (i.e. two authors independently involved to screen, extract or assess data in conduct of the review) for most review steps and narrative presentation of the results without a meta-analysis [41, 45, 47, 54, 58]. Scoping reviews tend not to provide standardised effect estimates, a quality assessment of individual evidence sources and usually omit further quantitative analyses such as sensitivity and subgroup analyses [9, 49]. Therefore, the global rating of CAT HPPR for a review depends on the assigned type of review (and, if applicable, the complementary review approach), the research question (C1) and the kind of data of included evidence sources extracted and used for the review (see C10). End-users need to first select the correct algorithm that applies to the appraised review and follow the instructions for each individual criterion regarding eligibility in the global rating process. The biggest difference between each review type in the global rating process lies in the fact that C9 and C10 are in some cases treated as “critical criteria” or are sometimes not treated as such (see Table 1). In stark contrast to a systematic review, some methodological aspects (four-eyes principle, quality assessment of included evidence sources) are often not defined as minimum requirements by (reporting) guidelines for reporting a rapid review or a scoping review (especially RRs), or in practice are simply not taken into consideration by review authors (especially ScRs) [9, 41, 45, 47, 49, 54, 55, 58].

### Shared experience made by CAT HPPR users

Based on user experience with CAT HPPR during the pilot phase, the processing time for an appraisal has been described as “manageable” and comparable to other CATs for systematic reviews [15]. Users needed approx. thirty minutes for a complete assessment run of a review manuscript with 40 pages, after they got familiar with the tool. End-users reported that reading the dictionary and all definitions of the manual for the first time, exceeded processing time of a review assessment and should be taken into consideration for introducing the tool to new users. All 14 review assessments during the pilot phase were successfully assessed with CAT HPPR, including final global ratings ranging from “very low” to “high” (with “moderate” being the least often reported global rating). This showcased the tool’s ability to differentiate between reviews of different quality: the tool ranked reviews with high reporting quality and appropriate use of standard review methods better than other reviews, which was later confirmed by oral feedback of end-users of the reports not directly involved in the CAT development process.

## Discussion

The development process of CAT HPPR led to a manual and appraisal form which can be now used by academic researchers, students and practitioners in health promotion and prevention to asses a variety of different review types and analytic designs. Existing CATs, especially AMSTAR 2 and ROBIS, which are well suited for assessing systematic reviews with intervention type studies and meta-analysis, remain relevant in academia to this day [15, 38] — developing CAT HPPR was not intended to replace them. In the case of healthevidence.org CAT, the tool has proven that ratings which are later added as supplementary information to a publicly available review database increase confidence in the evidence provided [14]. CAT HPPR fills the gap in case reviews of different types need to be assessed at the same time and introduces a strategy to decompose complexity by having different algorithms for achieving an informative, meaningful and yet nuanced global rating. A key strength of this work is that review types such as rapid and scoping reviews as well as mixed-methods reviews and review of reviews – so far overlooked by CAT developers – can be now assessed using a transparent method [13–15, 20, 24, 28–30, 33–38]. Prior to the development of CAT HPPR quality assessments of these review types had to rely on CATs originally designed for systematic reviews where many items do not capture the full breadth of other review types (e.g. as review of reviews: reviews and not primary studies as documents to be included; with mixed-methods-approach: integration of quantitative and qualitative data). We tried to overcome this challenge by using new reporting standards (e.g. PRISMA-ScR, guidelines by JBI) and our own working definitions for reviews to inform CAT HPPR [1, 2, 9]. Working in close partnership with experts in the field of meta research led to adjustments of CAT HPPR to make it more accessible for persons with no prior in-depth knowledge on reviews. The development process of CAT HPPR was not without limitations, given the short development time and opportunistic approach we took. In particular, we had hoped to test inter-rater reliability using κ scores for agreement between pairs of raters for assessing reviews of different formats which should be tested in future research [15]. The tool was also piloted with a limited set of commissioned reviews and the identification strategy for reporting guidelines, CATs, and items could have been improved by conducting a scoping review at the beginning, including a broader search. And despite using recent reporting guidelines and research to inform working definitions for review types and complementary review approaches, definitions of review types might still be subject of ongoing scientific discussion and also change over time. We tried to partly overcome this issue by letting the assessor decide, based on our guidance provided in the manual, that the assignment to a review type and complementary review approach does not necessarily have to correspond to the original label used by review authors to describe their own work (e.g. (i) authors’ label for review: “overview of reviews”; label used for the CAT HPPR assessment: “systematic review” “as review of reviews”). Lastly, for interpreting the global rating of a rapid review in particular, another factor should be cautiously factored in regarding the certainty of the evidence: using abridged review methods can by default result in the exclusion and/or non-consideration of relevant evidence in comparison to a systematic review. This similarly applies to scoping reviews, which sometimes are exclusively used to generate working definitions and to investigate boundaries of a research topic [5, 9]. Again, therefore, comparisons between different review types based on the global rating should be avoided (see Manual).

## Conclusions

The CAT HPPR can inform research and evidence based public health practice that aims to assess the quality of different review formats and analytic approaches. The tool pursues the dual aim of integrating existing knowledge of established CATs for systematic reviews (i.e. AMSTAR 2, healthevidence.org [14, 15]) with reporting standards as well as author guidance of emerging review formats (scoping reviews, rapid reviews) which became recently available [2, 5, 7–9, 41, 43–55]. To our knowledge, no similar CAT has been published to date that takes this unified approach allowing to assess reviews which include other “evidence sources” than traditional experimental clinical trials (e.g. RCTs). The available manual provides in-depth guidance on how to make objective assessments leading to a global rating across different review types. Criteria and definitions reported in the CAT HPPR manual might also help authors to improve their own work, by being aware of differences and standards for different review types and complementary approaches. We acknowledge one minor limitation. During development, piloting was conducted with an earlier version of the tool, therefore, inter-rater agreement should be investigated in future research. Nevertheless, we anticipate that CAT HPPR, including developed review definitions, will inform ongoing practice for EBPH- and implementation-oriented individuals and organisations. In particular, we stress the importance of using review evidence with high levels of transparency as well as methodological sound reporting and content. We call on researchers and practitioners alike to work with the CAT HPPR and welcome feedback for further development.

## Supplementary Information

**Additional file 1.**

**Additional file 2.**

## Data Availability

All documents for using the CAT HPPR are appended as online supplementary materials or directly included in this article as figures, tables or text. Additional data and documents used in development stages of the tool, including the research protocol, are available upon reasonable request. For this reason, please contact the corresponding author (Thomas L Heise).

## Abbreviations

AMSTAR: A MeaSurement Tool to Assess systematic Reviews
AMSTAR 2: Revision of AMSTAR
AQASR: Assessing the Quality and Applicability of Systematic Reviews
CAT: Critical Appraisal Tool
CAT HPPR: Critical Appraisal Tool for Health Promotion and Prevention Reviews
EQUATOR: Enhancing the QUAlity and Transparency Of health Research
GKV: Gesetzliche Krankenversicherung (German Statutory Health Insurance Funds)
JBI: Joanna Briggs Institute
MEDLINE: Medical Literature Analysis and Retrieval System Online (U.S. National Library of Medicine)
MECIR: Methodological Expectations of Cochrane Intervention Reviews
PCC: Population, Concept and Context
PICO(−TSSD): Population/patient/problem; Intervention, strategy or phenomenon of interest; Comparator; Outcomes, results of interest; Timing of outcome/follow-up measurement/assessment; Setting; Study-Design
PRISMA: Preferred Reporting Items for Systematic Reviews and Meta-Analyses
PRISMA-ScR: PRISMA Extension for Scoping Reviews
RCT: Randomized controlled trial
RoB: Risk of Bias
RR: Rapid Review
ScR: Scoping Review
SR: Systematic Review
SURE: Specialist Unit for Review Evidence
