# Supplementary material for: CAT HPPR: a critical appraisal tool to assess the quality of systematic, rapid, and scoping reviews investigating interventions in health promotion and prevention
Source: BMC Med Res Methodol. 2022 Dec 26;22:334. doi: 10.1186/s12874-022-01821-4 (PMC9791771; doi:10.1186/s12874-022-01821-4)
Supplement: Supplementary file 1 — Additional file 1. [file 12874_2022_1821_MOESM1_ESM.zip › CAT HPPR assessment form_EN.pdf]

CAT HPPR

Assessment form of the Critical Appraisal Tool for Health Promotion and Prevention Reviews (CAT HPPR)

First author:	
Year of publication:	
Article for appraisal<br>(full reference / source):	
Critical appraisal was conducted according to one of the following review types (optional: select a complementary review approach):	**Review type**<br>☐ Systematic Review<br>☐ Rapid Review<br>☐ Scoping Review<br>(only one answer possible)<br><br>**Complementary review approach of a review type**<br>☐ […] as Review of Reviews<br>☐ […] with Mixed-Methods-Approach<br>☐ […] with Meta-Analysis<br>(more than one answer possible)<br><br>☐ Appraisal not possible because article/report does not apply to any pre-specified review type or cannot be assigned
Filled out and appraised by:	
Global rating regarding the certainty in the methodological quality and results of the review:	☐ High<br>☐ Moderate<br>☐ Low<br>☐ Very Low<br>☐ Appraisal not possible (four (RR; ScR) or five (SR) or more critical criteria were assessed as not applicable (appraisable))<br>(only one answer possible)
Additional comments regarding the rating process:	General:<br>C1:<br>C2:<br>C3:<br>C4:<br>C5:<br>C6:<br>C7:<br>C8:<br>C9:<br>C10:<br>C11:<br>C12:<br>C13:<br>C14:<br>C15:

1. CAT HPPR assessment form: appraisal matrix

CRITERIA (CRITICAL)	YES	NO	NA[2]
**C1. Is the review based on a clear and focused question that has been adequately formulated and reported?**[1]<br>For documentation, please tick relevant boxes of concepts which were part of the question (PICO(-TSSD); PCC (see below)) and were reported in the section describing the objectives of the review:<br>☐ **P** Population/patient/problem<br>☐ **I** Intervention, strategy or phenomenon of interest<br>☐ **C** Comparator<br>☐ **O** Outcomes, results of interest<br>☐ **T** Timing of outcome/follow-up measurement/assessment<br>☐ **S** Setting<br>☐ **SD** Study design<br>(more than one answer possible)<br><br>For Scoping Reviews: assessment by means of other question frameworks (PCC) possible<br>☐ **P** Population<br>☐ **C** Concept<br>☐ **C** Context<br>(more than one answer possible)<br><br>[1] Inclusion criteria of C1 for global rating is further described in chapter 7	☐	☐	☐
**C2. Were methods of this review transparently reported prior to conduct of the review?**<br>For documentation, please tick relevant boxes of the documents in which the methodology of the review was described:<br>☐ Methods section of the report/article<br>☐ Published review protocol as journal article (e.g. journal with peer-review process)<br>☐ Published review protocol in database (e.g. PROSPERO, OSF)<br>☐ Review protocol available elsewhere (e.g. document of contractual arrangements)<br>(more than one answer possible)	☐	☐	☐
**C3. Were appropriate in- and exclusion criteria used in the selection process (title-/abstract and full text screening) of evidence sources (i.e. scientific work: studies, reviews, project reports, etc.)?**	☐	☐	☐

[2] NA (not applicable/no rating possible) (see chapter 3)

CRITERIA (CRITICAL)	YES	NO	NA[2]
**C4. Was a search strategy for databases and/or other sources of information reported by the authors which can be considered as comprehensive?**<br>For documentation, please tick relevant boxes of searched and reported databases and/or sources of information:<br>☐ Relevant, subject-specific bibliographic databases (e.g. MEDLINE, EMBASE, CINAHL, PsycINFO)<br>☐ Relevant, multidisciplinary bibliographic databases (e.g. Web of Science, Scopus, DOAJ)<br>(more than one answer possible)<br>______________________________<br>☐ Relevant bibliographic databases for grey literature (e.g. OpenGrey)<br>☐ Other specialised databases/registries (e.g. study registries, project databases)<br>☐ Relevant websites (e.g. professional associations, institutions)<br>☐ Reference lists (e.g. existing reviews on the topic, of included studies)<br>☐ Handsearches (e.g. manual searches in key journals or conference publications)<br>☐ Consultations with experts<br>☐ Unpublished literature (e.g. provided by authors)<br>☐ Citation tracking<br>(more than one answer possible)	☐	☐	☐
**C5. Was the selection process of evidence sources from search to synthesis transparently reported?**<br>For documentation, please tick relevant boxes of all documentation forms used that were reported to illustrate the selection process:<br>☐ Flowchart regarding the selection (e.g. PRISMA flow diagram)<br>☐ Presence of a descriptive (sub)chapter on the selection process<br>☐ Providing a list of excluded evidence sources (e.g. studies) with justification<br>(more than one answer possible)	☐	☐	☐
**C6. Is a description of characteristics of included evidence sources provided in the review (especially PICO(-TSSD) or PCC elements)?**<br>For documentation, please tick relevant boxes where authors provided information on characteristics (most commonly reported: study characteristics):<br>☐ Information in text<br>☐ Information in tables<br>(more than one answer possible)	☐	☐	☐
**C7. Were appropriate methods used to combine or compare the results of included evidence sources?**<br>For documentation, please tick relevant boxes for all methods that contributed to and were used for presenting main results:<br>☐ Narrative Synthesis<br>☐ Meta-Analysis<br>______________________________<br>☐ Data visualization approaches for evidence synthesis (e.g. forest plots, funnel plots, effect-direction plots, harvest plots etc.)<br>☐ Presentation in tabular form for reporting results of the evidence synthesis<br>☐ Presentation in main text for reporting results of the evidence synthesis<br>(more than one answer possible)	☐	☐	☐

[2] NA (not applicable/no rating possible) (see chapter 3)

CRITERIA (CRITICAL)	YES	NO	NA[2]
**C8. Do the results of the included evidence sources support the interpretation of the review authors?**	☐	☐	☐

CRITERIA (CRITICAL)[3]	YES	NO	NA[2]
CRITERIA (FOR ADDITIONAL DOCUMENTATION)[3]	YES	NO	NA[2]
**C9. Did the review team follow the four-eyes principle for important steps within the review process to reduce the risk of errors and biased decisions?**<br>For documentation, please tick relevant boxes for steps in the review process where the four-eyes principle was followed:<br>☐ In the selection of evidence sources: Title- and Abstract screening<br>☐ In the selection of evidence sources: Full text screening<br>☐ During data extraction<br>☐ In assessing the quality of evidence sources*<br>(more than one answer possible; * to be answered for reviews with a quality assessment)	☐	☐	☐
**C10. Was a methodological quality assessment of included evidence sources, based on established criteria or a tool, part of the review?**<br>For documentation, please tick relevant boxes for areas which were investigated in the quality assessment of included evidence sources:<br>☐ Study design<br>☐ Study sample (selection, generalizability, description of study population at baseline)<br>☐ Participation rates<br>☐ Data collection (measurement of independent/dependent variables)<br>☐ Follow-up/attrition rates<br>☐ Intervention integrity (intervention carried out as intended?)<br>☐ Estimation of exposure (information bias)<br>☐ Data analysis<br>☐ Other sources for potential bias (e.g. confounding variables, response bias, funding bias)<br>(more than one answer possible)<br><br>Alternative: For documentation, please tick the relevant box if an established quality assessment tool was used:<br>☐ Application of established quality assessment tool (e.g. Cochrane RoB, Cochrane RoB 2, Newcastle-Ottawa Scale, EPHPP QAT, JBI CATs, ROBIS, AMSTAR 2, COSMIN)<br>(one answer possible)	☐	☐	☐

[2] NA (not applicable/no rating possible) (see chapter 3)

[3] Inclusion criteria for C9-C10 to be included in the global rating process are reported in chapter 7.

CRITERIA (NON-CRITICAL)	YES	NO	NA[2]
**C11. Was the risk of bias of included evidence sources incorporated in the presentation and discussion of the review findings or were strengths and weaknesses of the evidence sources critically discussed?**<br>Tick the box indicating the form in which consideration was given:<br>☐ Systematic approach for rating the certainty of the evidence (i.e. GRADE)<br>☐ Considered within the synthesis<br>☐ Mentioned in the results section<br>☐ Mentioned in the discussion section<br>(more than one answer possible)	☐	☐	☐
**C12. Were homogeneity or heterogeneity of included evidence sources adequately considered in the review process and sufficiently presented in the final review?**<br>For documentation, please tick relevant boxes to indicate how homogeneity/heterogeneity of the evidence sources was considered and presented:<br>☐ Description to report similarities and differences of included evidence sources (especially with reference to the review's PICO(-TSSD) or PCC)<br>☐ By means of statistical tests for homogeneity/heterogeneity°<br>(more than one answer possible; ° to be answered for reviews with quantitative synthesis methods)	☐	☐	☐
**C13. Were methodological limitations of the selected review type and methods sufficiently addressed in the discussion?**	☐	☐	☐

CRITERIA (FOR ADDITIONAL DOCUMENTATION)	YES	NO	NA[2]
**C14. Were potential conflicts of interest (including funding) of the review authors provided in the review or actively declared as non-existing?**	☐	☐	☐
**C15. Were all relevant outcomes, including negative/adverse aspects of the object of consideration, mentioned?**<br>For documentation, please tick relevant boxes on whether negative aspects of the object under consideration were mentioned:<br>☐ As included outcome (in the methods section)<br>☐ Mentioned in the results section<br>☐ Mentioned in the discussion section<br>(more than one answer possible)<br><br>For documentation, please tick the relevant box if planned outcomes correspond to the final reported outcomes in the review:<br>☐ Planned outcomes correspond to reported outcomes in the result or discussion section<br>(one answer possible)	☐	☐	☐

[2] NA (not applicable/no rating possible) (see chapter 3)

GLOBAL RATING
**Global rating of the general confidence in the methodological quality and results of the review**
Based on your ratings for critical and non-critical categories, please tick the global rating for the assessed review:<br>☐ High<br>☐ Moderate<br>☐ Low<br>☐ Very low<br>☐ Appraisal not possible (four (RR; ScR) or five (SR) or more critical criteria were assessed as not applicable (appraisable)) (only one answer possible)
