# Supplementary material for: CAT HPPR: a critical appraisal tool to assess the quality of systematic, rapid, and scoping reviews investigating interventions in health promotion and prevention
Source: BMC Med Res Methodol. 2022 Dec 26;22:334. doi: 10.1186/s12874-022-01821-4 (PMC9791771; doi:10.1186/s12874-022-01821-4)
Supplement: Supplementary file 1 — Additional file 1. [file 12874_2022_1821_MOESM1_ESM.zip › CAT HPPR manual_EN.pdf]

CAT HPPR

Manual and instructions to reviewers for using the Critical Appraisal Tool for Health Promotion and Prevention Reviews (CAT HPPR)

24 July 2020

Edited by Thomas L Heise, Andreas Seidler, Maria Girbig, Alice Freiberg, Hajo Zeeb

Contents

List of abbreviations ... 2

Glossary ... 4

List of figures ... 7

List of tables ... 7

1. Background and objectives ... 8

2. Brief overview of the CAT HPPR development process ... 9

3. Introduction to CAT HPPR ... 9

4. CAT HPPR assessment form: summary page ... 12

5. CAT HPPR assessment form: appraisal matrix ... 13

6. CAT HPPR dictionary: reaching a judgement for a specific criterion ... 18

7. Structure of the global rating system by review type and inclusion of criteria ... 34

7.1 Global rating: Systematic Review ... 35

7.2 Global rating: Rapid or Scoping Review ... 37

Appendix 1: On the definition of review types ... 39

Systematic Review ... 39

Rapid Review ... 40

Scoping Review ... 40

[…] as Review of Reviews ... 41

[…] with Mixed-Methods-Approach ... 42

[…] with Meta-Analysis ... 43

References ... 43

List of abbreviations

**AMSTAR**	**A** **M**ea**S**urement **T**ool to **A**ssess systematic **R**eviews
**AMSTAR 2**	Revision of AMSTAR
**CAT**	Critical Appraisal Tool
**CAT HPPR**	Critical Appraisal Tool for Health Promotion and Prevention Reviews
**CINAHL**	Cumulative Index to Nursing and Allied Health Literature (EBSCO)
**Cochrane RoB**	Cochrane Risk of Bias Tool
**Cochrane RoB 2**	Revision of Cochrane RoB
**COSMIN**	**CO**nsensus-based **S**tandards for the selection of health **M**easurement **IN**struments
**DOAJ**	Directory of Open Access Journals
**Embase**	Excerpta Medica database (Elsevier)
**EPHPP QAT**	Effective Public Health Practice Project (EPHPP) Quality Assessment Tool for Quantitative Studies
**GRADE**	Grading of Recommendations Assessment, Development and Evaluation
**JBI**	Joanna Briggs Institute
**MEDLINE**	Medical Literature Analysis and Retrieval System Online (U.S. National Library of Medicine)
**MECIR**	Methodological Expectations of Cochrane Intervention Reviews
**NOS**	Newcastle-Ottawa Scale
**NICE**	National Institute for Health and Care Excellence
**OpenGrey**	System for Information on Grey Literature in Europe (INIST-CNRS)
**OSF**	Open Science Framework
**PCC**	**P**opulation, **C**oncept and **C**ontext
**PICO(-TSSD)**	**P**opulation/patient/problem; **I**ntervention, strategy or phenomenon of interest; **C**omparator; **O**utcomes, results of interest; **T**iming of outcome/follow-up measurement/assessment; **S**etting; **S**tudy-**D**esign
**PsycINFO**	Bibliographic database of the American Psychological Association
**PROSPERO**	International Prospective Register of Systematic Reviews (NIHR)

**PRISMA**	Preferred Reporting Items for Systematic Reviews and Meta-Analyses
**PRISMA-P**	Preferred Reporting Items for Systematic Reviews and Meta-Analyses Protocols
**PRISMA-ScR**	PRISMA Extension for Scoping Reviews
**RCT**	Randomized controlled trial
**ROBIS**	Tool designed to assess Risk of Bias in Systematic Reviews
**RoB**	Risk of Bias
**Scopus**	Abstract and citation database of Elsevier
**QAT**	Quality Appraisal Tool
**Web of Science**	Bibliographic database of Clarivate Analytics

Glossary

**Bayesian approaches to mixed-methods synthesis**	Bayesian integration of quantitative and qualitative data in research synthesis. A conversion of data (e.g. numerical value attributed to all qualitative data or codification of quantitative data) is often a basic requirement.
**Bias**	Systematic error or deviation in results or inferences from the truth or true values (e.g. selection bias: bias due to incorrect or inconsistent recruitment methods; information bias: bias due to incorrect or inaccurate collection, recall, recording or handling of information, etc.)
**Boolean operator**	Logical operators such as AND, OR and NOT used in most information databases to connect search terms or field searches.
**Cochrane**	International not-for-profit organisation which supports the production, revision and dissemination of so-called Cochrane Reviews containing credible health information.
**Cochrane Review**	Systematic summaries of evidence by Cochrane which are published in the Cochrane library and fulfil certain methodological expectations (MECIR standards). Their methodological approach is often regarded as the "gold standard" for Systematic Reviews.
**Confounder**	A factor or confounding variable that is associated with both an intervention (or exposure) and the observed outcome of interest.
**Data Charting**	Data extraction process in Scoping Reviews to inform a logical and descriptive summary which aligns with the objectives and questions of the review.
**Evidence**	General term for all research- and experience-based published or unpublished information that can be used as a source of information to decide whether a belief or proposition is true or valid and, thereby, is highly relevant to decision-making.
**Evidence source**	A generic term introduced for this project to describe scientific work (studies, reviews, project reports, other documents, records of registry databases) and other written documents that contain evidence and can therefore be included or excluded as a source of information in reviews.
**Effect-Direction-Plot**	Visual representation of effect direction - a standardised metric - for outcome domains across studies in synthesis without meta-analysis.
**Forest-Plot**	Graphical representation of the individual results of each evidence source included in a meta-analysis, along with the overall results.

**Funnel-Plot**	Scatter plot in which the effect size of different evidence sources is plotted against a measure of precision or other study information. It is commonly used within reviews with meta-analysis to check for biasing factors, such as reporting bias.
**Follow-up**	Observation period of a study after baseline measurement, during which the occurrence of events in the study population is recorded.
**Generalisability**	Extent to which findings from an evidence source provide a correct basis for generalisations to other circumstances such as other settings or populations.
**Grey literature**	Refers to documents, reports, discussion papers or other documents produced and published by governmental entities, academic institutions or other author groups. Compared to study articles published by established publishers, "grey literature" often requires more time and effort to be found. Furthermore, "grey literature" often does not undergo a regular peer review process before it is published.
**Harvest-Plot**	Method for presenting effects from complex and heterogeneous evidence sources in synthesis without meta-analysis.
**Conflicts of interest**	Opposing interests which can unduly influence professional judgement concerning a primary interest. A primary interest such as the validity of research can be consciously or unconsciously undermined by a secondary interest (e.g. financial gain via money from third-parties).
**Meta-analysis**	Use of statistical techniques for synthesizing the results of several evidence sources dealing with the same question.
**Peer-Review-Process**	A refereeing process for scientific reports. Other experts (e.g. scientists) review manuscripts prior to publication. It is usually carried out in multiple stages, which include a check for quality and importance.
**Title- and Abstract Screening**	Process which includes a systematic examination of titles and abstracts (e.g. of bibliographic records) to determine whether a particular evidence source is eligible for the review.
**Review**	Scientific report that summarises the current state of research for a particular topic or research field and, thereby, enables an effective examination of several evidence sources. (see: Appendix 1)
**Four-eyes principle**	Implies that for important decisions and steps in the review process (especially screening and data extraction) not only one person but at least two persons must be involved and, if possible, decisions should be made by consensus (or by involving a third person).

**Full text screening**	Process which includes a systematic examination of full texts to determine whether a particular evidence source is eligible for the review.
**Vote Counting**	Method used in synthesis without meta-analysis in which the number of studies regarding a direction of an effect is counted. Limitations result from the fact that the strength of the overall effect size cannot be reliably estimated and the study population size of individual studies is not (or only insufficiently) taken into account. (Other alternatives to meta-analysis, such as the Effect-Direction-Plot or Harvest-Plot, share similar limitations).

List of figures

Figure 1 Critical appraisal process using CAT HPPR 11

List of tables

Table 1 CAT HPPR assessment form: summary page for the assessed review 12
Table 2 CAT HPPR assessment form: appraisal matrix for the assessed review 17
Table 3 Inclusion of criteria in the global rating process 34
Table 4 Inclusion of criteria in the global rating process: Systematic Review 35
Table 5 Inclusion of criteria in the global rating process: Rapid or Scoping Review 37

1. Background and objectives

Reviews in health promotion and prevention research are used to summarise, analyse, and assess various evidence sources for answering a particular research question. The relevance of following internationally well-established review guidelines and standardised procedures in order to increase the quality of both reporting and applied methods and, thereby, content quality of reviews has been widely recognised by publishers as well as author groups in recent years [1-4]. In addition to this development a separate line of research, first mentioned in the medical context more than 30 years ago, has been established. The development and application of Critical Appraisal Tools (CATs) is devoted to explore ways of assessing the quality of reviews [5, 6]. Beyond being able to draw conclusions on the overall reporting quality of a review, the aim of using a CAT instrument is to assess as transparently and objectively as possible the selection and application of adequate review methods and to identify major methodological shortcomings or bias, including the appropriateness of review conclusions. Covering methodological aspects of different review sections, this process can lead to an overall rating regarding the general quality of a review report [7, 8]. Various CATs have been developed over time for different fields of application and target audiences, which mainly differ in the degree of manualisation (i.e. guidance documents with further explanations), type of questions (e.g. open or closed), type of answers (including the number of answer options) and the effort and time required for completion. Against this backdrop, the area of application of CATs can be further classified into scientific use cases [7, 9], education purposes [10, 11] or guideline development [12-14]. Further, assessments with CATs are helpful for end-users of review products such as practitioners and can be provided as supplementary information in a curated review database with a particular research scope [8]. This last use case represents the target scope of the Critical Appraisal Tool for Health Promotion and Prevention Reviews (CAT HPPR).

The “GKV-Bündnis für Gesundheit”, a joint initiative of all health insurance funds for developing and implementing setting-based health promotion and prevention measures, commissioned a series of reviews on strategies and interventions for health promotion and disease prevention. Final review reports were later compiled and made available to the interested public. The commissioned reviews covered a broad range of topics and focused on different evidence sources (e.g. studies, project descriptions etc.) to be included. Led by decisions on content or scope, the type of review (e.g. as Systematic Reviews) and methods selected by the author teams to meet the objective of each review differed somewhat [15]. This mix in applied review methodology can also be seen in reviews published by international journals in the field of health promotion and prevention. Existing CATs and their criteria are predominantly designed to assess Systematic Reviews, and were not well suited to reflect on and evaluate unique key aspects of some of the review types (e.g. Scoping Reviews) and complementary approaches (e.g. Mixed Methods) which were part of this review collection [7, 8]. More specifically, during our search for published CATs, we also could not identify CATs which were exclusively designed to be used for assessing Scoping Reviews or Rapid Reviews.

The lack of a well-documented CAT for various types of reviews motivated the development of this new tool. The goals of the project were thus: (i) to describe relevant types of reviews and complementary approaches in order to report working definitions for setting the target scope for the application of the CAT HPPR, (ii) to develop an innovative appraisal tool based on key criteria of existing CATs (e.g. healthevidence.org, AMSTAR 2) including a manual for end-users of the tool and (iii) to pilot the tool with a limited set of reviews from the German “GKV-Bündnis für Gesundheit”.

2. Brief overview of the CAT HPPR development process

For developing the CAT HPPR, we first used a pragmatic search of relevant websites (including the EQUATOR Network, Cochrane, JBI), electronic databases (e.g. Medline) and references provided by project partners in order to identify relevant reporting guidelines/standards (n=4) [1-4] as well as further guidance documents for conducting a review of a particular kind (Systematic Reviews, Rapid Reviews, Scoping Reviews) and also guidance on using complementary review approaches (as Review of Reviews, with Mixed-Methods-Approach, with Meta-Analysis) (n = 16) [16-31]. Based on identified documents, narrow working definitions were developed and further refined, involving all project partners (tool developers, members of a project-specific reviewer pool, on behalf of the GKV-Bündnis für Gesundheit: BZgA project partners). Because there is still a lack of consensus for labelling and a distinction between different types of reviews and their complementary approaches in the scientific literature, this step was crucial for achieving better applicability of the to-be-developed appraisal tool, its criteria and the global rating algorithm (see Appendix 1). Methodologically less narrowly defined types of reviews (e.g. Overviews) or those that had a very large overlap with the types and approaches we had already defined (e.g. Mapping Reviews) were not considered separately. As a further step towards identifying and tailoring relevant content of pre-existing CAT and their criteria for our tool, we carried out an electronic search using the same approach as we did for reporting standards. We assessed 27 full-texts of CATs and other review evaluation instruments for eligibility, 14 CATs were included as relevant [6-10, 12-14, 32-37], which were mainly developed for the quality assessment of Systematic Reviews. Since the CAT of healthevidence.org shared the most similar aim and content with our to-be-developed CAT, individual criteria of this tool were first extracted and compared to extracted criteria from the remaining 13 CATs. All content was translated into German language during this process. Translations were checked by a second project member. Extracted criteria with the same wording or content across different CATs/instruments were removed. After reviewing all criteria, discussion and consensus decisions of the project team, the number of 46 identified individual criteria was reduced to 15. Decisions for inclusion were based on the ability of each criterion to be comprehensive, relevant and objectively appraisable. We also used reporting guidelines/standards as well as guidance documents for developing reviews for setting basic requirements for each criterion to be fulfilled by a review and further guidance for users for reaching a judgement. Finally, after piloting a first version of the CAT HPPR with 14 reviews, feedback and requests for further clarification by users of the tool and experts of the project-specific reviewer pool led to final adjustments of the tool. Thus, a review-type specific algorithm was introduced in the global rating system in order to better take methodological advantages and disadvantages of individual review types into account. Among other things, the "Risk of Bias Assessment" was thus highlighted as a basic requirement and quality feature in Systematic Reviews compared to other review types.

3. Introduction to CAT HPPR

This manual provides further guidance and explanations on how to use the CAT HPPR, as each appraisal follows a standardised procedure which is outlined below. The overall aim is to fill out the CAT HPPR assessment form for each article or report under consideration, including justified ratings for each criterion and a global rating for the review. Please refer to the instructions below, including instructions for completing the individual sections of the assessment, as well as definitions and review types and potential complementary approaches used in a review (see Appendix 1).

Each assessment starts with an assignment to or definition of the review type (and, if applicable, the complementary review approach based on this type) which forms the basis for the entire appraisal process to come (see Appendix 1). This is a basic requirement so that each criterion and question can be rated or answered in accordance to the methodological requirements for a particular review type and, if applicable, approach. The assignment to a review type and complementary review approach **does not necessarily** have to correspond to the original label used by review authors to describe their own work (e.g. authors' label for review: "Overview of Reviews"; label used for the CAT HPPR assessment: "Systematic Review" "as Review of Reviews"). If this is the case, you can leave a comment under "General:" on the summary page.

Before using the CAT HPPR, please carefully read all the instructions provided in Chapter 6 (**the CAT HPPR dictionary**). This chapter provides useful information for reaching a judgement for a specific criterion and outlines which information needs to be available in the review. Here, the information in the text box serves as a guideline for reaching the final judgement. In addition to the obligatory consideration of "hard" criteria (i.e. information for reaching a judgement of **YES** or **NO**), further "soft" criteria (in parentheses (Note: …)) in a box offer a general orientation as to which factors may also influence the rating.

A rating with **YES** should be given in the majority of cases if information is available and/or if the requirement has been adequately fulfilled. Conversely, a rating with **NO** is usually given in cases of missing information and/or a largely incorrect implementation or use with regards to reliable review procedures and methods. If the answer to a single question is not possible due to a mismatch between the question and the review in question, your answer should neither be expressed as NO nor YES; in this case please tick the box **NA (not applicable/no rating possible)** instead. In general, ratings should be reported by placing a check mark (or cross) in the appropriate box. This also applies to sub-items that are listed under each question. By providing these further documentation options, this should lead to a more transparent decision process. Under "Additional comments regarding the rating process", you can note additional comments regarding reaching your judgement, including relevant page numbers of the review providing information to each question, especially if a rating was not possible. Based on user experience with CAT HPPR during a pilot phase, the processing time for an appraisal is manageable. Users needed approx. 30 minutes for a complete assessment run of a review manuscript with 40 pages, after they got familiar with the tool, including reading the dictionary and all definitions.

A **global rating** is based on the rating results for **critical** and **non-critical** criteria and follows the logic described in chapter 7. Make sure that the correct algorithm for the review type is being used. Transfer the global rating to the summary page. "Criteria (for additional documentation)" as defined in chapter 7 do not contribute to the global rating. A global rating **cannot** be achieved in two cases: (i) If a decision based on Appendix 1 has been made beforehand that a critical appraisal is not possible because the article or report does not correspond to any of the listed review types or could not be assigned to one or (ii) if four or more critical criteria have been judged to be **NA**. In the first case, only the summary page of the assessment form has to be completed.

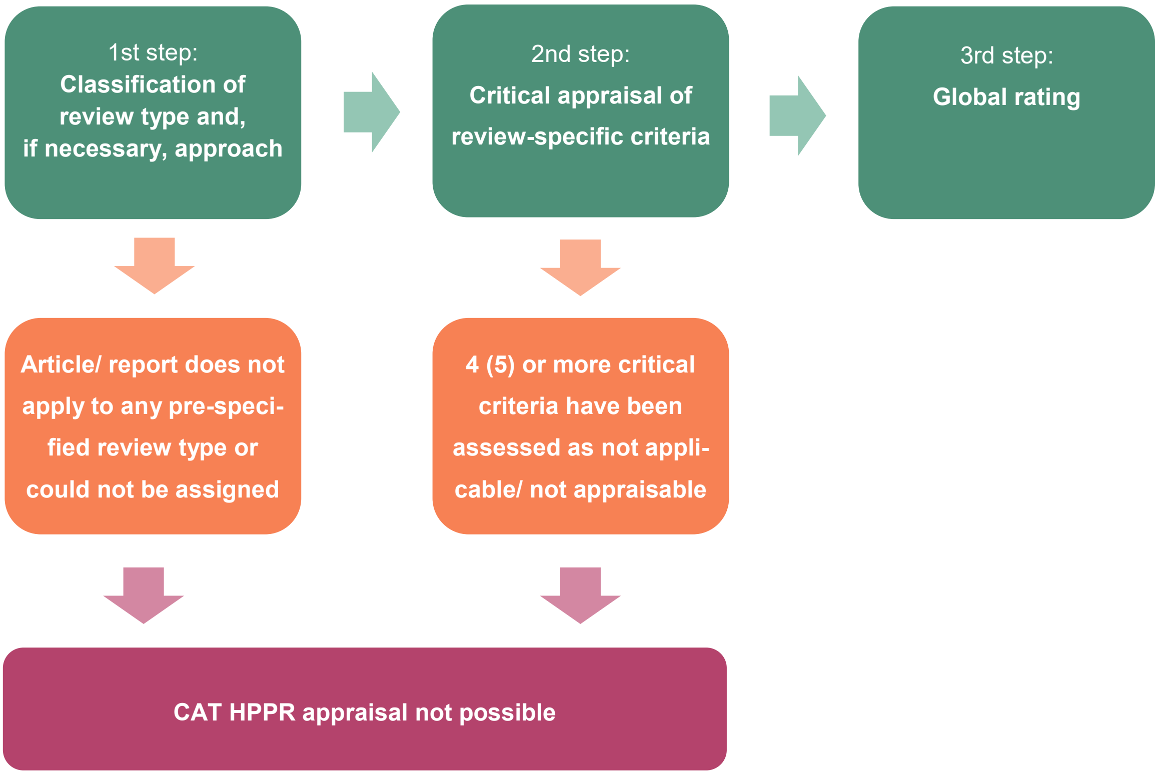

Figure 1 Critical appraisal process using CAT HPPR

4. CAT HPPR assessment form: summary page

First author:	
Year of publication:	
Article for appraisal<br>(full reference / source):	
Critical appraisal was conducted according to one of the following review types (optional: select a complementary review approach):	**Review type**<br>☐ Systematic Review<br>☐ Rapid Review<br>☐ Scoping Review<br>(only one answer possible)<br><br>**Complementary review approach of a review type**<br>☐ […] as Review of Reviews<br>☐ […] with Mixed-Methods-Approach<br>☐ […] with Meta-Analysis<br>(more than one answer possible)<br><br>☐ Appraisal not possible because article/report does not apply to any pre-specified review type or cannot be assigned
Filled out and appraised by:	
Global rating regarding the certainty in the methodological quality and results of the review:	☐ High<br>☐ Moderate<br>☐ Low<br>☐ Very Low<br>☐ Appraisal not possible (four (RR; ScR) or five (SR) or more critical criteria were assessed as not applicable (appraisable))<br>(only one answer possible)
Additional comments regarding the rat-ing process:	General:<br>C1:<br>C2:<br>C3:<br>C4:<br>C5:<br>C6:<br>C7:<br>C8:<br>C9:<br>C10:<br>C11:<br>C12:<br>C13:<br>C14:<br>C15:

Table 1 CAT HPPR assessment form: summary page for the assessed review

5. CAT HPPR assessment form: appraisal matrix

CRITERIA (CRITICAL)	YES	NO	NA[2]
**C1. Is the review based on a clear and focused question that has been adequately formulated and reported?**[1]<br>For documentation, please tick relevant boxes of concepts which were part of the question (PICO(-TSSD); PCC (see below)) and were reported in the objective section of the review:<br>☐ **P** Population/patient/problem<br>☐ **I** Intervention, strategy or phenomenon of interest<br>☐ **C** Comparator<br>☐ **O** Outcomes, results of interest<br>☐ **T** Timing of outcome/follow-up measurement/assessment<br>☐ **S** Setting<br>☐ **SD** Study design<br>(more than one answer possible)<br><br>For Scoping Reviews: assessment by means of other question frameworks (PCC) possible<br>☐ **P** Population<br>☐ **C** Concept<br>☐ **C** Context<br>(more than one answer possible)<br><br>[1] Inclusion criteria of C1 for global rating is further described in chapter 7	☐	☐	☐
**C2. Were methods of this review transparently reported prior to conduct of the review?**<br>For documentation, please tick relevant boxes of the documents in which the methodology of the review was described:<br>☐ Methods section of the report/article<br>☐ Published review protocol as journal article (e.g. journal with peer-review process)<br>☐ Published review protocol in database (e.g. PROSPERO, OSF)<br>☐ Review protocol available elsewhere (e.g. document of contractual arrangements)<br>(more than one answer possible)	☐	☐	☐
**C3. Were appropriate in- and exclusion criteria used in the selection process (title-/abstract and full text screening) of evidence sources (i.e. scientific work: studies, reviews, project reports, etc.)?**	☐	☐	☐

[2] NA (not applicable/no rating possible) (see chapter 3)

CRITERIA (CRITICAL)	YES	NO	NA[2]
**C4. Was a search strategy for databases and/or other sources of information reported by the authors which can be considered as comprehensive?**<br>For documentation, please tick relevant boxes of searched and reported databases and/or sources of information:<br>☐ Relevant, subject-specific bibliographic databases (e.g. MEDLINE, EMBASE, CINAHL, PsycINFO)<br>☐ Relevant, multidisciplinary bibliographic databases (e.g. Web of Science, Scopus, DOAJ) (more than one answer possible)<br>______________________________<br>☐ Relevant bibliographic databases for grey literature (e.g. OpenGrey)<br>☐ Other specialised databases/registries (e.g. study registries, project databases)<br>☐ Relevant websites (e.g. professional associations, institutions)<br>☐ Reference lists (e.g. existing reviews on the topic, of included studies)<br>☐ Handsearches (e.g. manual searches in key journals or conference publications)<br>☐ Consultations with experts<br>☐ Unpublished literature (e.g. provided by authors)<br>☐ Citation tracking<br>(more than one answer possible)	☐	☐	☐
**C5. Was the selection process of evidence sources from search to synthesis transparently reported?**<br>For documentation, please tick relevant boxes of all documentation forms used that were reported to illustrate the selection process:<br>☐ Flowchart regarding the selection (e.g. PRISMA flow diagram)<br>☐ Presence of a descriptive (sub)chapter on the selection process<br>☐ Providing a list of excluded evidence sources (e.g. studies) with justification<br>(more than one answer possible)	☐	☐	☐
**C6. Is a description of characteristics of included evidence sources provided in the review (especially PICO(-TSSD) or PCC elements)?**<br>For documentation, please tick relevant boxes where authors provided information on characteristics (most commonly reported: study characteristics):<br>☐ Information in text<br>☐ Information in tables<br>(more than one answer possible)	☐	☐	☐
**C7. Were appropriate methods used to combine or compare the results of included evidence sources?**<br>For documentation, please tick relevant boxes for all methods that contributed to and were used for presenting main results:<br>☐ Narrative Synthesis<br>☐ Meta-Analysis<br>____________________<br>☐ Data visualization approaches for evidence synthesis (e.g. forest plots, funnel plots, effect-direction plots, harvest plots etc.)<br>☐ Presentation in tabular form for reporting results of the evidence synthesis<br>☐ Presentation in main text for reporting results of the evidence synthesis<br>(more than one answer possible)	☐	☐	☐

[2] NA (not applicable/no rating possible) (see chapter 3)

CRITERIA (CRITICAL)	YES	NO	NA[2]
**C8. Do the results of the included evidence sources support the inter-pretation of the review authors?**	☐	☐	☐

CRITERIA (CRITICAL)[3]	YES	NO	NA[2]
CRITERIA (FOR ADDITIONAL DOCUMENTATION)[3]	YES	NO	NA[2]
**C9. Did the review team follow the four-eyes principle for important steps within the review process to reduce the risk of errors and biased decisions?**<br>For documentation, please tick relevant boxes for steps in the review process where the four-eyes principle was followed:<br>☐ In the selection of evidence sources: Title- and Abstract screening<br>☐ In the selection of evidence sources: Full text screening<br>☐ During data extraction<br>☐ In assessing the quality of evidence sources*<br>(more than one answer possible; * to be answered for reviews with a quality assessment)	☐	☐	☐
**C10. Was a methodological quality assessment of included evidence sources, based on established criteria or a tool, part of the review?**<br>For documentation, please tick relevant boxes for areas which were investigated in the quality assessment of included evidence sources:<br>☐ Study design<br>☐ Study sample (selection, generalizability, description of study population at baseline)<br>☐ Participation rates<br>☐ Data collection (measurement of independent/dependent variables)<br>☐ Follow-up/attrition rates<br>☐ Intervention integrity (intervention carried out as intended?)<br>☐ Estimation of exposure (information bias)<br>☐ Data analysis<br>☐ Other sources for potential bias (e.g. confounding variables, response bias, funding bias)<br>(more than one answer possible)<br><br>Alternative: For documentation, please tick the relevant box if an established quality as-sessment tool was used:<br>☐ Application of established quality assessment tool (e.g. Cochrane RoB, Cochrane RoB 2, Newcastle-Ottawa Scale, EPHPP QAT, JBI CATs, ROBIS, AMSTAR 2, COSMIN)<br>(one answer possible)	☐	☐	☐

[2] NA (not applicable/no rating possible) (see chapter 3)

[3] Inclusion criteria for C9-C10 to be included in the global rating process are reported in chapter 7.

CRITERIA (NON-CRITICAL)	YES	NO	NA[2]
**C11. Was the risk of bias of included evidence sources incorporated in the presentation and discussion of the review findings or were strengths and weaknesses of the evidence sources critically discussed?**<br>Tick the box indicating the form in which consideration was given:<br>☐ Systematic approach for rating the certainty of the evidence (i.e. GRADE)<br>☐ Considered within the synthesis<br>☐ Mentioned in the results section<br>☐ Mentioned in the discussion section<br>(more than one answer possible)	☐	☐	☐
**C12. Were homogeneity or heterogeneity of included evidence sources adequately considered in the review process and sufficiently presented in the final review?**<br>For documentation, please tick relevant boxes to indicate how homogeneity/heterogeneity of the evidence sources was considered and presented:<br>☐ Description to report similarities and differences of included evidence sources (especially with reference to the review's PICO(-TSSD) or PCC)<br>☐ By means of statistical tests for homogeneity/heterogeneity°<br>(more than one answer possible; ° to be answered for reviews with quantitative synthesis methods)	☐	☐	☐
**C13. Were methodological limitations of the selected review type and methods sufficiently addressed in the discussion?**	☐	☐	☐

CRITERIA (FOR ADDITIONAL DOCUMENTATION)	YES	NO	NA[2]
**C14. Were potential conflicts of interest (including funding) of the review authors provided in the review or actively declared as non-existing?**	☐	☐	☐
**C15. Were all relevant outcomes, including negative/adverse aspects of the object of consideration, mentioned?**<br>For documentation, please tick relevant boxes on whether negative aspects of the object under consideration were mentioned:<br>☐ As included outcome (in the methods section)<br>☐ Mentioned in the results section<br>☐ Mentioned in the discussion section<br>(more than one answer possible)<br><br>For documentation, please tick the relevant box if planned outcomes correspond to the final reported outcomes in the review:<br>☐ Planned outcomes correspond to reported outcomes in the result or discussion section<br>(one answer possible)	☐	☐	☐

[2] NA (not applicable/no rating possible) (see chapter 3)

GLOBAL RATING
**Global rating of the general confidence in the methodological quality and results of the review**
Based on your ratings for critical and non-critical categories, please tick the global rating for the assessed review:<br>☐ High<br>☐ Moderate<br>☐ Low<br>☐ Very low<br>☐ Appraisal not possible (four (RR; ScR) or five (SR) or more critical criteria were assessed as not applicable (appraisable))<br>(only one answer possible)

Table 2 CAT HPPR assessment form: appraisal matrix for the assessed review

6. CAT HPPR dictionary: reaching a judgement for a specific criterion

C1. Is the review based on a clear and focused question that has been adequately formulated and reported?

Each review should be based on a clear and focused research question. The **PICO(-TSSD)** question format provides guidance as to whether this has been sufficiently and successfully addressed by a review team conducting a systematic or rapid review. It covers the following aspects: **P** Population/patient/problem, **I** Intervention, strategy or phenomenon of interest, **C** Comparator and **O** Outcomes, results of interest; further, for more specific questions: **T** Timing of outcome/follow-up measurement/assessment, **S** Setting and **SD** Study design. Inclusion criteria should generally align with the review question. If in-/exclusion criteria are used to further narrow down the question (especially **C**, **O**, **T**, **S**, and **SD**), this should be justified by the authors in the review methods in order to answer C1 with **YES**. For example, a review question containing **P**, **I**, **O** and **S** elements was used by Goudet et al: "To assess the impact of nutritional interventions to reduce stunting in infants and children under five years old in urban slums from LMIC and the effect of nutritional interventions on other nutritional (wasting and underweight) and non-nutritional outcomes (socioeconomic, health and developmental) in addition to stunting". The authors of this Cochrane Review then used in-/exclusion criteria to further specify PICO(-TSSD) elements that were not yet addressed. Not every aspect of a PICO(-TSSD) question format is always ideal and relevant for conceptualising a review question (especially reviews that do not consider the effectiveness of an intervention or consider exposure) [38]. The **PCC** question format provides an alternative for conceptualising, documenting and assessing a review question and is often used for Scoping Reviews, especially for those that were not exclusively designed to produce results regarding a **PICO(-TSSD)**-oriented question. It covers some broader aspects, namely, **P** Population, **C** Concept and **C** Context. **P** Population generally has the same meaning as **P** Population has within the PICO question format. The part of a question that is used for describing a planned investigation of certain (methodological) aspects of an **I** intervention or an **O** Outcome or of the validity and reliability of an instrument can be considered as a **C** Concept in this regard. [17] However, the **C** concept used in a Scoping Review does not necessarily have to be related to an intervention and may instead be linked to research designs, frameworks, theories or classification systems, etc. [18] **C** Context shares similarities with **S** Setting of the extended PICO question format. **C** Context can thus be used e.g. for geographical, cultural, migration-related or gender-related specifications within the research question. If a Scoping Review has been assessed whilst using **PCC** concept in C1, the concept should be also considered for all other sections which are influenced by the review's main question (especially C3, C6 and C12).

→ Box "Coding for C1"

Coding for C1
(Note: Inclusion criteria for C1 to be included in the global rating process are reported in chapter 7; please proceed with the rating of C1 and the documentation of your judgement nonetheless).

Review type
□ **Systematic Review:** a review which investigated exposure or the effectiveness of an intervention has to cover all four aspects of the PICO question format in the review question (or by means of further specification and substantiated explanation within in-/exclusion criteria) in order to receive a **YES** [7, 8]. Otherwise, please tick the box in the **NO** column for those reviews. Reviews that did not investigate exposure or the effectiveness of an intervention should not be rated, since these questions do not allow a fair comparison regarding the question as reviews using the PICO format do. In this case, please tick **NA**.

□ **Rapid Review:** analogous to the assessment of a Systematic Review (Note: the question can be more specific).

□ **Scoping Review:** in the case of reaching a judgement using the PICO question format: analogous to the assessment of a Systematic Review. If using the PCC question format: all three aspects of a PCC question format have to be covered (within the question or by means of further specification and substantiated explanation within in-/exclusion criteria) for an answer with **YES**. Otherwise, please tick the box in the **NO** column for those reviews. Reviews that did not deal with a question involving a population (missing P) should not be rated, since these questions do not allow a fair comparison regarding the question as reviews using the PCC format do. In this case, please tick **NA**. (Note: the question may be broader in scope).

Complementary review approach
□ **[…] as Review of Reviews:** your original assessment based on the review type applies. Furthermore, it must be clearly stated in the research question (or by means of further specification within in-/exclusion criteria) that reviews were considered as the source of information. Otherwise, please answer with **NO**.

□ **[…] with Mixed-Methods-Approach:** your original assessment based on the review type applies. Furthermore, it must be clearly stated in the research question (or by means of further specification and substantiated explanation within in-/exclusion criteria) that quantitative and qualitative studies were considered as a source of information. Otherwise, please tick the box in the **NO** column.

□ **[…] with Meta-Analysis:** your original assessment based on the review type applies.

C2. Were methods of this review transparently reported prior to conduct of the review?

In order to fulfil the function of offering a ‘higher’ level of evidence as compared to findings of an individual evidence source, reviews should, as required for individual studies, be based on a protocol which details in advance the review’s rationale, objective and methods. Following a well-described protocol while conducting the review reduces the risk of bias (e.g. in- or exclusion of evidence sources contrary to pre-defined eligibility criteria). The gold standard for reviews is a published protocol in a

journal with a peer-review-process meeting the requirements of an appropriate reporting guideline (e.g. PRISMA-P) [2]. Moreover, registering a review protocol in a specialized register such as PROSPERO (https://www.crd.york.ac.uk/PROSPERO) or OSF (https://osf.io/) provides the opportunity of communicating amendments, changes and updates throughout the entire review process which fosters transparency and reproducibility [7]. Reviews (often Rapid Reviews or Scoping Reviews), which are developed in close consultation and partnership with commissioning organisations or funding entities, should at least describe the negotiation process and final agreements made regarding the review's objective and methods in the case of a non-published protocol, so that comparisons with the initial research plan, idea or call can be drawn.

→ Box "Coding for C2"

Coding for C2

Review type

□ **Systematic Review:** A review must (i) have included a citation or reference to a previously developed review protocol outlining its methods (e.g. active record on a register such as PROSPERO or OSF, as a separate journal article, contractual document, a clear reference to protocol development prior to conduct of the review in the review document (i.e. with reference to the negotiations concerning review components and methods between authors and commissioners)) and (ii) in case of obvious or serious methodological differences between the protocol and the review, have sufficiently reported and justified these in order to receive a **YES**. If either no information indicating a pre-developed review protocol or with reference to key pre-developed methods is provided or if obvious differences between the protocol and the review have not sufficiently been reported, please tick the box **NO**. [7]

□ **Rapid Review:** analogous to the assessment of a Systematic Review. (Note: especially with reference to the negotiations concerning review components and methods between authors and commissioners if a study protocol has not been published).

□ **Scoping Review:** analogous to the assessment of a Systematic Review. (Note: especially with reference to the negotiations concerning review components and methods between authors and commissioners if a study protocol has not been published).

Complementary review approach

□ **[…] as Review of Reviews:** your original assessment based on the review type applies.

□ **[…] with Mixed-Methods-Approach:** your original assessment based on the review type applies.

□ **[…] with Meta-Analysis:** your original assessment based on the review type applies.

C3. Were appropriate in- and exclusion criteria used in the selection process (title-/abstract and full text screening) of evidence sources (i.e. scientific work: studies, reviews, project reports, etc.)?

The review should have clearly reported in its methods section eligibility criteria by which the included evidence sources were selected and non-relevant sources were excluded. Decisions must have been related to aspects of the PICO(-TSSD) question format, in particular the target population, the

intervention, the strategy or the phenomenon of interest, the outcomes (including measurement approaches or instruments) and the study/research design. The latter can relate, for example, to randomized (or cluster-randomized) controlled trials, quasi-randomized controlled trials, non-randomized controlled trials, controlled before-after studies, uncontrolled before-after studies, studies with interrupted time series design, historically controlled studies, and studies using a defined qualitative research approach. The exclusive use of rather vague descriptions such as "studies that underwent any type of peer-review-process" and/or "studies with quantitative outcome assessment" does not adequately represent a study design of interest. In the case that authors exclusively reported which study design they had excluded, but did not specifically explain which study designs they actually had included, please tick **NO** for this criterion. [8] For Scoping Reviews with PCC question format, the definition of in- and exclusion criteria must have been defined along aspects of the PCC format for receiving a **YES**. [17]

→ Box "Coding for C3"

Coding for C3

Review type

□ **Systematic Review:** Answer **YES** if reported in- and exclusion criteria were clearly defined along relevant aspects of the PICO(-TSSD) format and were sufficiently justified. If they are not clearly described, please tick **NO**.

□ **Rapid Review:** analogous to the assessment of a Systematic Review. (Note: this may apply in particular to geographical or language restrictions regarding, or comparisons, outcomes, study/research design and publication date of an evidence source).

□ **Scoping Review:** analogous to the assessment of a Systematic Review. In Scoping Reviews with a PCC question format, in- and exclusion criteria must have been defined along relevant PCC elements to receive a **YES**. (Note: this may apply in particular to criteria for evidence sources that were in- or excluded on the basis of the study/research design, type of report or data).

Complementary review approach

□ **[…] as Review of Reviews:** your original assessment based on the review type applies. (Note: this may apply in particular to the type of review, which was in- or excluded as part on decisions for an eligible study design).

□ **[…] with Mixed-Methods-Approach:** your original assessment based on the review type applies. (Note: this may apply in particular to the separate presentation of in- and exclusion criteria for quantitative and qualitative evidence sources for research/study designs under consideration).

□ **[…] with Meta-Analysis:** your original assessment based on the review type applies. (Note: this may apply in particular to the description of eligible comparisons, timing of outcome measurement/follow-up, outcomes (including measurement approaches or instruments)).

C4. Was a search strategy for databases and/or other sources of information reported by the authors which can be considered as comprehensive?

A well-documented and comprehensive search strategy includes search approaches for multiple literature databases and other search streams to identify relevant evidence sources. The selection of subject-specific (e.g. MEDLINE, EMBASE, CINAHL, PsycINFO) and/or non-subject-specific/multidisciplinary bibliographic databases (e.g. Web of Science) should be based on the focus of the review question. Reporting of a complete search syntax (typically provided for one database) facilitates the understanding of possible limitations, the width and correctness of a search [2]. Other search streams regarding information sources can especially help to identify 'grey literature' (i.e. literature that is not controlled or published by commercial or established academic publishers), unpublished literature and documents/records of ongoing projects/studies. Search techniques such as citation tracking and searching reference lists also help to identify evidence sources not identified through original database searches and, if applicable, compensate for insufficient indexing or potential non-listing of relevant literature in databases.

→ Box "Coding for C4"

Coding for C4

Review type

□ **Systematic Review:** For answering with **YES**, the authors must have searched at least three relevant bibliographic databases, which can be subject-specific and/or non-subject-specific/multidisciplinary [4]. At least the syntax of one search (including logical operators, filters etc.) must be described in its entirety (in the protocol or in the final review document or its appendix). In addition to searching academic databases, the authors must have used at least two other search approaches (e.g. grey literature databases, other types of databases (especially study registers), relevant websites, reference lists, handsearches, consultation with experts, unpublished literature, citation tracking). If applicable, a justification for using search restrictions (especially search limits affecting the time period covered) must be provided to receive a **YES**. [1, 4, 8]

□ **Rapid Review:** To answer with **YES**, the search must have been performed in at least one literature database and by using another search approach (e.g. searches based on reference lists) or by conducting a search in a second database. [19]

□ **Scoping Review:** analogous to the assessment of a Rapid Review.

Complementary review approach

□ **[…] as Review of Reviews:** your original assessment based on the review type applies.

□ **[…] with Mixed-Methods-Approach:** your original assessment based on the review type applies.

□ **[…] with Meta-Analysis:** your original assessment based on the review type applies.

C5. Was the selection process of evidence sources from search to synthesis transparently reported?

The selection process, which is usually performed in two stages (title-/abstract and full text screening), can best be described as a funnel in which relevant evidence sources (e.g. relevant articles) are gradually being included and non-relevant sources are excluded. Since this two step-process involves decisions made by the authors along pre-defined eligibility criteria and, thereby, excluded evidence sources are consciously regarded as irrelevant for answering the specific review question—the only logical conclusion is a comprehensive documentation. In the first, much broader step of the selection process (based on the title and the abstract), for reasons of efficiency, a breakdown of reasons for exclusion is usually not provided. However, in the second step of screening full texts a detailed breakdown of reasons which led to exclusions is required by report guidelines such as PRISMA [4]. In general, the selection process should be presented in the text and/or as a flowchart [4]. Using the flowchart as a reference point helps to consider important aspects and stages of the screening and review process.

→ Box "Coding for C5"

Coding for C5

Review type

□ **Systematic Review:** Answer **YES**, if sufficient information regarding the search and selection process of evidence sources is provided in the format of a complete PRISMA flow diagram or core aspects of it have been narratively reported, i.e:

- Search hits retrieved from database searches or other sources,
- Total number of records after removing duplicates,
- Number of in- and excluded records after screening titles and abstracts, as well as after full text screening; for full text screening: additional breakdown of reasons which led to exclusions (desirable but not obligatory: additional lists of excluded evidence sources with reference and reason for exclusion),
- Number of evidence sources included in the final review (desirable but not obligatory: number of evidence sources included in the narrative or statistical synthesis).

If the information provided in the flowchart does not match the information in the text, or if no PRISMA flow diagram or a comprehensive narrative description of the selection process (see above) has been reported, please tick **NO**. [1]

□ **Rapid Review:** analogous to the assessment of a Systematic Review.

□ **Scoping Review:** analogous to the assessment of a Systematic Review.

Complementary review approach

□ **[…] as Review of Reviews:** your original assessment based on the review type applies.

□ **[…] with Mixed-Methods-Approach:** your original assessment based on the review type applies. A breakdown by the number of quantitative and qualitative evidence sources must have been described in a comprehensible manner (e.g. in the flowchart or the presentation of characteristics of the included evidence sources in text or tables) in order to obtain a **YES**.

□ **[…] with Meta-Analysis:** your original assessment based on the review type applies. A breakdown by the number of evidence sources included in the Meta-analysis must have been described in a comprehensible manner in order to mark **YES**.

C6. Is a description of characteristics of included evidence sources provided in the review (especially PICO(-TSSD) or PCC elements)?

The description of characteristics of included evidence sources can be part of the results (e.g. in Scoping Reviews) as well as an intermediate step informing the synthesis of a review. Tabular presentation of this kind of data is particularly useful to provide a comparative overview covering key aspects which are essential to the research question. In general, only relevant study characteristics should be reported in order to maintain clarity, thereby, enabling the assessment of relevancy and homogeneity or heterogeneity of the included data by readers. [1, 3, 8]

→ Box "Coding for C6"

**Coding for C6**<br><br>Review type<br>□ **Systematic Review:** Please answer with **YES**, if the study characteristics (or characteristics of the included evidence sources) were sufficiently presented in text and/or tables so that end-users (like you) can make an independent assessment of whether the included evidence sources can adequately contribute information regarding the original review question and whether the in- and exclusion criteria were sufficiently followed by the authors in the screening process (see C3). To this end, a presentation of the characteristics along PICO(-TSSD) elements often remains a key requirement. Please answer **NO**, if a review does not sufficiently provide information on characteristics of included evidence sources (e.g. studies).<br><br>□ **Rapid Review:** analogous to the assessment of a Systematic Review. (Note: the content and overall presentation as text or tables can be shorter).<br><br>□ **Scoping Review:** analogous to the assessment of a Systematic Review. For Scoping Reviews with a question following the PCC concept, it is also useful if authors reported characteristics along major PCC elements.<br><br>Complementary review approach<br>□ **[…] as Review of Reviews:** your original assessment based on the review type applies. Key characteristics of the included reviews (e.g. date range covered, number of studies) should have been reported in order to receive a **YES**.<br><br>□ **[…] with Mixed-Methods-Approach:** your original assessment based on the review type applies.<br><br>□ **[…] with Meta-Analysis:** your original assessment based on the review type applies. (Note: In order to draw a conclusion on whether all relevant studies have been included in the meta-analysis at a later stage, review authors must have mentioned all the outcomes which had data available in the individual evidence sources that were in line with the inclusion criteria of the review before).

C7. Were appropriate methods used to combine or compare the results of included evidence sources?

Depending on the included evidence sources and the research question of interest, different approaches are available for synthesising the results (or data) in the review (see Appendix 1). In general, established review methods can be found in published guidelines by JBI, NICE and Cochrane [17, 18, 20, 39, 40] and should be considered prior to conduct of the review. A meaningful appraisal of applied methods for the synthesis is only possible if authors have outlined their methodological synthesis approach in the methods section of the protocol or final review, especially with regard to the question of whether basic requirements for a certain systematic synthesis approach are met. Depending on the review type and complementary review approach, different methodological approaches should be used [17, 18]. In the event of heterogeneous evidence sources included, e.g. due to differences in included study designs, it should be considered prior to conduct whether evidence sources of different kinds can be meaningfully synthesised or whether they should be treated separately in the synthesis. The description in the box below provides some advice for the appraisal.

→ Box "Coding for C7"

Coding for C7

Review type
□ **Systematic Review:** Answer **YES**, if authors provided at least a well-balanced narrative synthesis considering all included evidence sources (presented in tables and/or text). The synthesis approach must be the same as outlined in the methods section of the review and, if applicable, as pre-defined in the protocol. If only selective and unsystematic reporting of individual evidence sources becomes apparent (i.e. not all relevant references identified through the screening process were considered in the synthesis), or if limitations (which may arise from the selected synthesis approach (especially so-called "vote counting")) were not acknowledged by the authors, please tick **NO**. [41]

□ **Rapid Review:** analogous to the assessment of a Systematic Review.

□ **Scoping Review:** analogous to the assessment of a Systematic Review.

Complementary review approach
□ **[…] as Review of Reviews:** your original assessment based on the review type applies. In this case, a **NO** is also possible if authors have performed a quantitative synthesis (e.g. meta-analysis) on an aggregated level without accounting for the entry of data from the same original data source (i.e. reviews with a huge overlap in included studies were included in the quantitative synthesis) [21].

□ **[…] with Mixed-Methods-Approach:** To answer with **YES**, a description must be provided of whether analyses (i) were first performed separate from each other or in parallel according to the included qualitative and quantitative data and its structure and then integrated into a mixed methods synthesis ("segregated synthesis"), or (ii) a combined approach for qualitative and quantitative evidence sources was used (e.g. by a subsequent data conversion into a comparable format) (assimilated data; "integrated synthesis"), or (iii) a sequential analysis based on each other ("contingent synthesis") was conducted [22]. If no uniform synthesis approach (Note: The synthesis approach may have been labelled differently by the authors) was used in the review, please tick **NO**.

□ **[…] with Meta-Analysis:** For **YES**, authors did explain why they had performed the Meta-analysis and used an appropriate method for calculating weights of individual evidence sources. If known methodological limitations (e.g. combined synthesis of adjusted and non-adjusted values) remain uncommented by the authors or obvious methodological errors exist (e.g. same data from the same data source reported in different articles were considered multiple times in one Meta-analysis), **NO** should be ticked [7, 8].

C8. Do the results of the included evidence sources support the interpretation of the review authors?

Check the data of evidence sources included as reported by the review authors, especially in the results section, and decide whether they support the reviewers' interpretation. For a review with no statistical synthesis approach, or substantial levels of observed heterogeneity, or only a limited number of evidence sources included, the interpretation and formulation of findings should be cautious and the uncertainty of the results should be at the forefront of the interpretation - this also applies to shared recommendations by the authors. For sections which generally respond to key findings and are subject to the reviewers' interpretation, it should also be avoided to mainly refer to sources that have not gone through the systematic screening and selection process of the review. [42]

→ Box "Coding for C8"

**Coding for C8**
Review type<br>□ **Systematic Review:** To answer with <u>**YES**</u>, the interpretation by the review authors has to be in line with data of the included evidence sources. If the interpretation of the results is inappropriate, for example authors make a causal claim (A causes B, instead of using a more cautious interpretation), but the (summarized and synthesized) evidence sources do not confirm this cause and effect relationship or the methodological approach does not allow causal reasoning (e.g. no Meta-Analysis of RCTs, lack of statistical significance, "vote counting" within the synthesis), <u>**NO**</u> should be ticked [42]<br><br>□ **Rapid Review:** analogous to the assessment of a Systematic Review.<br><br>□ **Scoping Review:** analogous to the assessment of a Systematic Review.<br><br>Complementary review approach<br>□ **[…] as Review of Reviews:** your original assessment based on the review type applies.<br><br>□ **[…] with Mixed-Methods-Approach:** your original assessment based on the review type applies.<br><br>□ **[…] with Meta-Analysis:** your original assessment based on the review type applies.

C9. Did the review team follow the four-eyes principle for important steps within the review process to reduce the risk of errors and biased decisions?

Best practice for selecting studies, assessing the quality and extracting data (if available) is to follow the four-eyes principle which can help to avoid errors, biased decisions and, thereby, contribute to the overall decision quality [4]. Ideally, the extent of agreement can be tested by means of inter-rater reliability (e.g. kappa coefficient) during a pilot-phase or at each review step, to determine whether decision-relevant criteria have been clearly defined or understood [7]. Since assessing and reporting these numbers is time-consuming, it is rarely used and reported at the moment despite its relevance and is, therefore, currently not to be considered for assessing C9.

→ Box "Coding for C9"

Coding for C9
(Note: Inclusion criteria for C9 to be included in the global rating process are reported in chapter 7; please proceed with the rating of C9 and the documentation of your judgement nonetheless).

Review type
□ **Systematic Review:** All steps in selecting evidence sources (i.e. title/abstract and full text screening) and the quality assessment process (if available) have to be performed by at least two people independently and extracted data needs to be reviewed by a second person to receive a **<u>YES</u>**. In addition, a description should be provided on how disagreements in the study selection, data extraction and quality assessment process were handled (e.g. involvement of a third person). [4]

□ **Rapid Review:** analogous to the assessment of a Systematic Review.

□ **Scoping Review:** analogous to the assessment of a Systematic Review.

Complementary review approach
□ **[…] as Review of Reviews:** your original assessment based on the review type applies.

□ **[…] with Mixed-Methods-Approach:** your original assessment based on the review type applies.

□ **[…] with Meta-Analysis:** your original assessment based on the review type applies.

C10. Was a methodological quality assessment of included evidence sources, based on established criteria or a tool, part of the review?

Assessing potential bias of the included evidence by conducting a quality assessment can be considered as a review result on its own and helps to further understand the certainty of the evidence. If a systematic approach is used to determine the certainty of the evidence (see C11), such as GRADE, a methodological quality assessment of studies is obligatory [8]. Conducting a quality assessment enables drawing conclusions regarding the heterogeneity of included study results. The quality assessment tool or approach should be selected according to study designs or data included in the final review. Here, study design specific (e.g. AMSTAR 2 for a review conducted as a Review of Reviews [7]) or cross-study design approaches (e.g. EPHPP QAT for reviews including different study designs [43]) can be considered by the review team. However, for some review questions, it is generally not possible to assess the quality of the evidence by the use of an established tool. Nevertheless, while reaching a judgement for C10, it should be considered whether individual components as described on the assessment form can contribute to an overall positive rating (e.g. quality assessments regarding study design, data analysis or other sources for potential bias).

→ Box "Coding for C10"

Coding for C10
(Note: Inclusion criteria for C10 to be included in the global rating process are reported in chapter 7; please proceed with the rating of C10 and the documentation of your judgement nonetheless).

Review type
□ **Systematic Review:** (i) To answer with **YES**, the document under consideration needs to be a review of quantitative studies and at least four of the nine criteria described in the documentation of the appraisal matrix must have been assessed and ratings have been reported. (ii) If an established and appropriate quality assessment tool (including Cochrane RoB, Cochrane RoB 2, Newcastle-Ottawa Scale, EPHPP QAT, JBI CATs, ROBIS, AMSTAR 2, COSMIN) has been properly applied and the results of the quality assessment have been reported, the answer should be **YES**. In most other cases please tick **NO**. A review with qualitative studies, where a positive or negative assessment for C10 is currently not advised, should lead to tick the box **NOT APPLICABLE** [44].

□ **Rapid Review:** analogous to the assessment of a Systematic Review.

□ **Scoping Review:** analogous to the assessment of a Systematic Review.

Complementary review approach
□ **[…] as Review of Reviews:** analogous to the assessment as described under (ii) for a Systematic Review. (Note: The quality assessment is often done by using AMSTAR (2), but another established CAT may also indicate a **YES**).

□ **[…] with Mixed-Methods-Approach:** regarding the quality assessment of quantitative studies included in the review: analogous to the assessment of a Systematic Review.

□ **[…] with Meta-Analysis:** your original assessment based on the review type applies.

C11. Was the risk of bias of included evidence sources incorporated in the presentation and discussion of the review findings or were strengths and weaknesses of the evidence sources critically discussed?

Authors of a review should generally consider the impact of a quality assessment (if available) or generally consider strengths and weaknesses of the synthesised evidence in the interpretation of the review findings. This is particularly important when different study designs were included or in presence of a high variability in quality assessment results among individual evidence sources or, in a broader sense, after weighing up strengths and weaknesses of included evidence sources. [7]

→ Box "Coding for C11"

Coding for C11

Review type

□ **Systematic Review:** To answer with **<u>YES</u>**, the impact of any type of quality assessment regarding the evidence sources must have been included in the interpretation of the review results (especially in formulating recommendations for practice). If information which stems from the quality assessment was not mentioned in the results and the discussion sections, please answer with **<u>NO</u>** [7]. In special cases: In order to answer with **<u>YES,</u>** at least methodological strengths and weaknesses of the evidence sources in the results and discussion sections were reported, if a quality assessment was not possible/had not been carried out.

□ **Rapid Review:** For **<u>YES</u>**, at least methodological strengths and weaknesses of the evidence sources in the results and discussion sections were reported.

□ **Scoping Review:** analogous to the assessment of a Rapid Review.

Complementary review approach

□ **[…] as Review of Reviews:** your original assessment based on the review type applies.

□ **[…] with Mixed-Methods-Approach:** your original assessment based on the review type applies.

□ **[…] with Meta-Analysis:** For **<u>YES</u>**, the impact of any type of quality assessment regarding the evidence sources must have been included in the interpretation of pooled effect estimates (especially in formulating recommendations for practice). A systematic approach for rating the certainty of the evidence (i.e. GRADE) is also desirable, but not mandatory for answering with **<u>YES</u>**. If none of the two requirements are met, please tick **<u>NO</u>** [4].

C12. Were homogeneity or heterogeneity of included evidence sources adequately considered in the review process and sufficiently presented in the final review?

It is important that (results from) evidence sources are checked for homogeneity (both statistically and narratively) before and within the evidence synthesis. For this purpose, tests of homogeneity or heterogeneity are statistical techniques that are widely used. In Meta-analyses, general considerations and test results towards heterogeneity should guide, for example, the approach of analysing the data (e.g. random effects vs. fixed effect model), which can influence the relative weights of the individual study estimate within a Meta-Analysis. For Scoping Reviews, where a statistical analysis/synthesis is not always possible or sometimes would lead to unmeaningful findings, the results or characteristics of evidence sources should be presented in graphical and tabular form in order to provide further useful information on similarity across included evidence sources. In general, if this information on key characteristics and the summarized results is not reported or presented inconsistently or conclusions on similarity cannot be drawn please answer with **<u>NO</u>** [8].

→ Box "Coding for C12"

Coding for C12

Review type

□ **Systematic Review:** For **YES**, characteristics of included evidence sources have been reported, are in line with inclusion/exclusion criteria (e.g. for a review question on children populations, only studies among children (or included as subpopulation) should have been included in the review) (see C6) and results regarding relevant outcomes must have been reported in a consistent and comprehensible manner (narrative and/or graphical form). Considerations, such as the fact that RCTs (with the potential to draw causal conclusions) often have smaller sample sizes compared to large-scale cohort studies does not automatically lower their information value, must have been taken into account while comparing study results (e.g. reported separately within the synthesis).

□ **Rapid Review:** analogous to the assessment of a Systematic Review.

□ **Scoping Review:** analogous to the assessment of a Systematic Review.

Complementary review approach

□ **[…] as Review of Reviews:** your original assessment based on the review type applies.

□ **[…] with Mixed-Methods-Approach:** your original assessment based on the review type applies. (Note: Special attention should be paid to similarity across qualitative evidence sources).

□ **[…] with Meta-Analysis:** your original assessment based on the review type applies. To answer **YES**, statistical heterogeneity tests must have been carried out (e.g. I^2, χ^2) and the test results must have been critically discussed. If this information is not available and/or has not been discussed and/or was misinterpreted (i.e. insufficient number of studies affecting the degrees of freedom for appropriate interpretation), please answer with **NO**. [8]

C13. Were methodological limitations of the selected review type and methods sufficiently addressed in the discussion?

Limitations in conduct of a review may be due to review type and complementary review approach selection, the search for and availability/provision of evidence sources, and do not end at the choice for a synthesis approach. Amongst other factors, these limitations may stem from external factors (e.g. funding of the review project) and the context of the research (e.g. topic has not been covered before). A critical reflection regarding decisions made in the review process is necessary to assess the uncertainty of the overall review results and to identify aspects of what future research should investigate. [33]

→ Box "Coding for C13"

Coding for C13

Review type
□ **Systematic Review:** To answer with **<u>YES</u>**, at least methodological aspects of the search for evidence sources (e.g. database selection) and the closely related topic of in- and exclusion criteria (e.g. included study designs) must have been critically discussed and reported with regard to potential impacts on the summarised evidence. In addition, reporting guidelines or standards which have been followed (i.e. PRISMA [1]) and possible changes of methods from research outline or protocol to review should have been addressed. (Note: The last two aspects are not always part of the discussion section and may not apply here, e.g. no deviations from protocol to review; aspects are discussed elsewhere in the review manuscript).

□ **Rapid Review:** To answer with **<u>YES</u>**, at least methodological aspects of the search for evidence sources (e.g. database selection) and the closely related topic of in- and exclusion criteria (e.g. included study designs) must have been critically discussed and reported with regard to potential impacts on the summarised evidence. In addition, known limitations caused by an application of abbreviated review methods (e.g. time period covered, single screening (by one person), no quality assessment of included evidence sources) should have been discussed. Possible changes from reported review methods (e.g. as outlined in the methods section) to review should have been explained [19]. (Note: The last aspects are not always part of the discussion section and may not apply here, e.g. no deviations from research outline to review; aspects are discussed elsewhere in the review manuscript).

□ **Scoping Review:** To answer with **<u>YES</u>**, at least methodological aspects of the search for evidence sources (e.g. database selection) and the closely related topic of in- and exclusion criteria (e.g. included study designs) must have been critically discussed and reported with regard to potential impacts on the summarised evidence. In addition, reporting guidelines or standards which have been followed (i.e. PRISMA-ScR, JBI[3, 17]) and possible changes from reported review methods (e.g. as outlined in the methods section, protocol or research outline) to review should have been addressed. (Note: The last aspects are not always part of the discussion section and may not apply here, e.g. no deviations from research outline to review; aspects are discussed elsewhere in the review manuscript).

Complementary review approach
□ **[…] as Review of Reviews:** your original assessment based on the review type applies.

□ **[…] with Mixed-Methods-Approach:** your original assessment based on the review type applies.

□ **[…] with Meta-Analysis:** your original assessment based on the review type applies. In addition, possible methodological limitations due to methods and data used for conducting the Meta-Analysis must have been addressed. (Note: This is especially true of missing data, where data imputation methods were potentially applied)

C14. Were potential conflicts of interest (including funding) of the review authors provided in the review or actively declared as non-existing?

Conflicts of interest affecting a review team (authors) can sometimes, not automatically, lead to biased decisions in conduct of a review, which can ultimately translate into biased review results. In this context, dependencies caused by financial or non-financial benefits can lead to a situation of conflicting interests (own vs. third-party interests) among authors. An adequate disclosure of potential conflicts of interest with regard to project funding sources or by declaring any existing or non-existing personal conflicts of interest provides the opportunity to create transparency for end-users of the review. In

general, this information helps to understand whether a review in question could potentially be negatively affected by any conflicts of interest. [7]

→ Box "Coding for C14"

Coding for C14
(Note: C14 is not included in the global rating process as outlined in chapter 7; please proceed with the rating of C14 and the documentation of your judgement nonetheless).

Review type
□ **Systematic Review:** Please answer with **YES,** if information regarding a potential funding source of the review or any other potential conflicts of interests have been disclosed with regard to the review. The required information can be expressed in two different ways, by declaring the existence of potential conflicts of interest or by declaring that no conflicts of interest are known or present. A review with no information available should lead to a rating with **NO**. (Note: The potential impact of funding bias which may affect included evidence sources is discussed in C10 and should not be considered here).

□ **Rapid Review:** analogous to the assessment of a Systematic Review.

□ **Scoping Review:** analogous to the assessment of a Systematic Review.

Complementary review approach
□ **[…] as Review of Reviews:** your original assessment based on the review type applies.

□ **[…] with Mixed-Methods-Approach:** your original assessment based on the review type applies.

□ **[…] with Meta-Analysis:** your original assessment based on the review type applies.

C15. Were all relevant outcomes, including negative/adverse aspects of the object of consideration, mentioned?

Review authors should remain as objective as possible whilst taking both positive and negative aspects as well as unintended or adverse effects of the research object under consideration into account. This requires that all planned outcomes (and their results) are reported throughout all relevant sections of the review. This is particularly true for the results and discussion section, even if findings are unclear, negative or unexpected. If outcomes are reported selectively (e.g. only significant results are reported), this can (un)intentionally contribute to a biased overall picture of review findings. [4, 37]

→ Box "Coding for C15"

Coding for C15
(Note: C15 is not included in the global rating process as outlined in chapter 7; please proceed with the rating of C15 and the documentation of your judgement nonetheless).

Review type
□ **Systematic Review:** For **YES**, all planned outcomes, as reported in the protocol and/or methods section, were mentioned in the results and discussion section. If there are serious deviations and/or the investigation of negative aspects/unintended effects of the research object under consideration based on the included evidence sources were neither originally planned nor reported, please answer with **NO** [4]. A statement which includes for example that an expected adverse effect of an intervention was considered as a relevant outcome prior to conduct of the review, but no studies with relevant data were identified should translate into a positive rating. If outcomes were not part of the actual research question (see C1), a rating with **NOT APPLICABLE** should be considered.

□ **Rapid Review:** For **YES**, all planned outcomes, as reported in the protocol and/or methods section, were mentioned in the results and discussion section. If outcomes were not part of the actual research question (see C1), a rating with **NOT APPLICABLE** should be considered.

□ **Scoping Review:** analogous to the assessment of a Rapid Review [23].

Complementary review approach
□ **[…] as Review of Reviews:** your original assessment based on the review type applies.

□ **[…] with Mixed-Methods-Approach:** your original assessment based on the review type applies. (Note: Findings from qualitative evidence sources can be used in particular for an explanation of negative aspects/unintended effects of the research object under consideration).

□ **[…] with Meta-Analysis:** your original assessment based on the review type applies. If Meta-Analyses on negative aspects/unintended effects were originally planned, but not carried out despite sufficient data being available, as shown in the review, a rating with **NO** should be considered.

7. Structure of the global rating system by review type and inclusion of criteria

A global rating for a review depends on the assigned type of review (and, if applicable, the complementary review approach), the research question (C1) and the kind of data of included evidence sources extracted and used for the review (see C10). Please first select the correct algorithm that applies to the appraised review and follow the instructions and footnotes for each individual criterion regarding eligibility in the global rating process. The biggest difference between each review type in the global rating process lies in the fact that C9 and C10 are in some cases treated as "critical criteria" or are sometimes not treated as such (see SR vs. RR, ScR). In stark contrast to a Systematic Review, some methodological aspects (four-eyes principle, quality assessment of included evidence sources) are often not defined as minimum requirements by (reporting) guidelines for reporting a Rapid Review or a Scoping Review (especially RRs), or in practice are simply not taken into consideration by review authors (especially ScRs). As a consequence, the number of criteria considered in the global rating can vary across different review types (see Table 1) so that comparability between global ratings of different reviews is only given within the same review type category. For interpreting the global rating of a Rapid Review in particular, another factor should be cautiously factored in regarding the certainty of the evidence: using abridged review methods can by default result in the exclusion and/or non-consideration of relevant evidence in comparison to a Systematic Review. This similarly applies to Scoping Reviews, which were primarily used to generate working definitions and to investigate boundaries of a research topic (see Appendix 1).

		**Maximum (minimum) number of criteria considered in the global rating**		**Number of non-considered criteria in the global rating**
		critical	non-critical	for additional documentation
**Review type**	SR	**10 (8)**	**3**	**2**
	RR, ScR	**8 (7)**	**3**	**4**

Table 3 Inclusion of criteria in the global rating process

7.1 Global rating: Systematic Review

		Criteria (critical, non-critical, for additional documentation)														
		C1	C2	C3	C4	C5	C6	C7	C8	C9	C10	C11	C12	C13	C14	C15
**Review type**	SR	X[1]	X	X	X	X	X	X	X	X	X[2]	X	X	X	X	X
**Complementary review approach**	RoR	X[1]	X	X	X	X	X	X	X	X	X	X	X	X	X	X
	MM	X[1]	X	X	X	X	X	X	X	X	X[2]	X	X	X	X	X
	MA	X[1]	X	X	X	X	X	X	X	X	X[2]	X	X	X	X	X

Table 4 Inclusion of criteria in the global rating process: Systematic Review

Further explanations

X = Included in the global rating process.
X = Either inclusion or non-consideration in the global rating process is further specified in the corresponding footnote.
X = Not included in the global rating process and used for additional documentation only.

Abbreviations
SR: Systematic Review; RoR: Review of Reviews; MM: Mixed-Methods-Approach; MA: Meta-Analysis

Footnotes

[1] Review, which has investigated the effectiveness of an intervention or an exposure.
[2] Review with 'quantitative' evidence sources included.

Global rating of the general confidence in the methodological quality and results of the Systematic Review

High

A maximum of one **non-critical** weakness and no **critical** weakness: the Systematic Review gives a thorough and extensive overview on the review topic based on the available evidence which is considered to be relevant. [7] Moreover, the reporting quality is high.

Moderate

Two **non-critical** weaknesses and no **critical** weakness: the Systematic Review probably gives a thorough overview on the review topic based on the available evidence which is considered to be relevant, but shows potential for improvement. Limitations and uncertainties, for example, could be better addressed in the reporting of the review.

Low

One **critical** weakness with or without other **non-critical** weaknesses [7]; or three **non-critical** weaknesses: due to limitations, indicated by one critical weakness, the Systematic Review may not provide a thorough overview on the review topic based on the available evidence which is considered to be relevant [7]. For a global rating based on three non-critical weaknesses, it is very likely that there is considerable potential for improvement for addressing limitations and uncertainties in the reporting of the review.

Very low

Two or more **critical** weaknesses with or without other **non-critical** weaknesses: the Systematic Review has considerable methodological limitations. Most likely the Systematic Review does not give a thorough overview on the review topic based on the available evidence.

Critical appraisal not possible

Five or more **critical** criteria were judged to be not applicable or not appraisable. This means that no reliable conclusions can be drawn regarding the general confidence in the methodological quality and results of the Systematic Review.

7.2 Global rating: Rapid or Scoping Review

		Criteria (critical, non-critical, for additional documentation)														
		C1	C2	C3	C4	C5	C6	C7	C8	C9	C10	C11	C12	C13	C14	C15
**Review-Format**	RR, ScR	X[1,2]	X	X	X	X	X	X	X	X	X	X	X	X	X	X
**Complementary review approach**	RoR	X[1,2]	X	X	X	X	X	X	X	X	X	X	X	X	X	X
	MM	X[1,2]	X	X	X	X	X	X	X	X	X	X	X	X	X	X
	MA	X[1,2]	X	X	X	X	X	X	X	X	X	X	X	X	X	X

Table 5 Inclusion of criteria in the global rating process: Rapid or Scoping Review

Further explanations

X = Included in the global rating process.

X = Either inclusion or non-consideration in the global rating process is further specified in the corresponding footnote.

X = Not included in the global rating process and used for additional documentation only.

Abbreviations

RR: Rapid Review; ScR: Scoping Review; RoR: Review of Reviews; MM: Mixed-Methods-Approach; MA: Meta-Analysis

Footnotes

[1] Review, which has investigated the effectiveness of an intervention or an exposure.

[2] Scoping Review, which contains a PCC-oriented question. This specification is not applicable to Rapid Reviews.

Global rating of the general confidence in the methodological quality and results of a Rapid or Scoping Review

High

A maximum of one **non-critical** weakness and no **critical** weakness: the Rapid or Scoping Review gives a thorough and extensive overview on the review topic based on the collected evidence. [7] Moreover, the reporting quality is high.

Moderate

Two **non-critical** weaknesses and no **critical** weakness: the Rapid or Scoping Review probably gives a thorough and extensive overview on the review topic based on the collected evidence, but shows potential for improvement. Limitations and uncertainties, for example, could be better addressed in the reporting of the review.

Low

One **critical** weakness with or without other **non-critical** weaknesses [7]; or three **non-critical** weaknesses: due to limitations, indicated by one critical weakness, the Rapid or Scoping Review may not provide a thorough overview on the review topic based on the collected evidence. For a global rating based on three non-critical weaknesses, it is very likely that there is considerable potential for improvement for addressing limitations and uncertainties in the reporting of the review.

Very low

Two or more **critical** weaknesses with or without other **non-critical** weaknesses: the Rapid or Scoping Review has considerable methodological limitations. Most likely the Rapid or Scoping Review does not give a thorough overview on the review topic based on the collected evidence.

Critical appraisal not possible

Four or more of the **critical** criteria were judged to be not applicable or not appraisable. This means that no reliable conclusions can be drawn regarding the general confidence in the methodological quality and results of the Rapid or Scoping Review.

Appendix 1: On the definition of review types

Systematic Review

A Systematic Review as a review type comprises a clear and focused research question and uses systematic and explicit methods to identify, select and critically assess relevant research or studies and aims to further analyse collected data of included studies or evidence sources. If extracted data of included studies or evidence sources are sufficiently available and methodologically as well as statistically homogeneous, further statistical approaches (Meta-analyses) can be used in later analysis and synthesis of the data (see: [...] with Meta-Analysis). [1] Working steps as well as documentation in conduct of a Systematic Review follow established guidelines (especially PRISMA, MECIR) in order to improve transparency of review findings and to strengthen the ability for a later replication or update of the review [2, 4, 17, 20].

Key characteristics of a Systematic Review

- Protocol (e.g. active record on PROSPERO, published article or contractual document): Published protocol (document outlining review methods a priori) which includes a clear and focused question along PICO(-TSSD) (population/patient/problem; intervention, strategy or phenomenon of interest; comparator; outcomes, results of interest; timing of outcome/follow-up measurement/assessment; setting; study-design) elements, in- and exclusion criteria, search strategy including information on selected databases, additional search approaches and selection process (title-/abstract as well as full text screening), data to be extracted, quality assessment tool to be used for included evidence sources, and information on systematic synthesis approach. Deviations from protocol to review reported and justified in the final review, if applicable. [1, 4, 17, 20]
- Outcome selection: outcomes also on possible negative aspects of the object of consideration (e.g. adverse effects of the intervention), as required by Cochrane standards, considered. [4]
- Search strategy: searches in multiple databases (Cochrane: at least three) and other search approaches used (e.g. study registers, reference lists, consultation with experts). At least syntax of one search entirely described (e.g. logical operators, filters) in the final report with date of the last search being reported [4]. Search limits affecting languages and the time period covered to be avoided, if possible and applicable.
- Selection process: complete flowchart documenting the selection process (e.g. PRISMA flow diagram). [1]
- Study characteristics: reported in a consistent and comprehensible manner (e.g. as text or in tabular form). [1, 4, 17, 20]
- Study designs: ranking order of study designs to be considered as evidence follow general recommendations to avoid potential bias (e.g. RCTs with commonly more reliable evidence compared to cohort studies, designs with control groups considered). [4]
- Four-eyes principle followed during screening, data extraction and quality assessment tasks while resolving potential conflicts by involving a third person. [20]
- Piloting prior to screening, data extraction and quality assessment. [4]
- Discussion section acknowledges methodological limitations. [1, 4, 20]

Rapid Review

Rapid reviews systematically summarise knowledge and are a review type in which the steps of a Systematic Review are abbreviated and/or accelerated (e.g. by using automated text mining and analysis processes) in order to provide evidence in a shorter production time (typically: 1 to 4 months [19]). [45] Rapid reviews share core elements of traditional Systematic Reviews, including a clear objective of the review, the definition of in- and exclusion criteria, an assessment of or critical reflection on the validity of the results (in some cases by assessing the risk of bias), and a systematic presentation and summary of review findings. Most commonly Rapid Reviews feature a reduced scope of the research question (including possible adjustments done multiple times during the review project), a reduction in number of search strings searched and use of further search limiters (e.g. time period covered, language restrictions), the omission of the four-eyes principle for most review steps and narrative presentation of the results without a Meta-analysis contributing to the review findings [19, 24, 25, 31, 45]. Reviews that can benefit from an increased use of (human) resources to lower the overall production time, but do not otherwise show any methodological shortcomings compared to traditional Systematic Review methods, should, strictly speaking, not be labelled as Rapid Reviews.

Key differences compared to core elements that lay the foundation for Systematic Reviews

- Methods section of the review or review protocol (e.g. active record on PROSPERO or contractual document): much narrowly defined PICO(-TSSD) oriented question (population/patient/problem; intervention, strategy or phenomenon of interest; comparator; outcomes, results of interest; timing of outcome/follow-up measurement/assessment; setting; study-design). [19]
- Search strategy: search in at least one database in combination with another search approach (e.g. reference lists of included studies) or a second database. [46]
- Alternative to following the four-eyes principle: screening, data extraction and quality assessment is carried out by an experienced review author, but at least one other person randomly checks votes, data and assessments. [19]
- Discussion section acknowledges methodological limitations, which arise in particular from applying abbreviated review methods (e.g. reduced scope of search strategy etc.). [27]

Scoping Review

Scoping Reviews are used as systematic knowledge synthesis to gather existing evidence on a topic and to identify and present main concepts, theories, evidence sources and research gaps in a comprehensible manner. Scoping Reviews are often undertaken in complex research contexts and research fields where no review is available and/or the research topic is new or innovative. [3, 18] It can also be used to shed light on how the research was actually conducted (e.g. research methods) [18, 23]. Compared to more traditional Systematic Reviews, Scoping Reviews tend not to provide standardised effect estimates, a quality assessment of individual evidence sources and omit further quantitative analyses such as sensitivity and subgroup analyses [3, 23]. Findings of a Scoping Review, however, can be the starting point of a more focused and comprehensive Systematic Review - especially when decisions for eligibility criteria are unclear prior to conduct of a Systematic Review - and can also bring up new research ideas by identifying research gaps and needs [28, 29]. The search strategy for Scoping Reviews usually accounts for multiple search streams, so Scoping Reviews often include heterogeneous evidence sources or data (e.g. quantitative and/or qualitative research, expert opinions, guidelines, strategy papers, project reports, economic evaluations etc.) (see: [...] with Mixed-Methods Approach) [3, 18]. The process of extracting data in Scoping Reviews is sometimes called "data charting" (i.e. a logical

and descriptive overview of the results arranged along objectives and research questions of the Scoping Review) [3, 17], which can later inform the final review findings presented within text, tables or charts (e.g. thematic analyses, summary of concepts) [3, 17]. Many Scoping Reviews are still conducted without adhering to the four-eyes principle in major review work packages for now [26]. However, guidelines specifically developed for Scoping Reviews show increased popularity amongst researchers (in particular JBI, PRISMA-ScR) which may translate into increased future uptake of this principle; a favourable outcome, which could later shift to a much stronger recognition of this principle within the quality assessment of Scoping Reviews [3, 17].

Key differences compared to core elements that lay the foundation for Systematic Reviews

- Methods section of the review or review protocol (e.g. active record on OSF, website, contractual document): clear, but usually broadly defined questions, especially in case of a population, concept and context (PCC) oriented question, broader in- and exclusion criteria, especially a wider breadth of evidence sources and data sources included, and different methods for processing data during synthesis. [3, 28]
- Study designs: evidence sources need to be grouped consistently in the presentation of results [17]. For Scoping Reviews, usually no restrictions regarding study designs are required.
- Synthesis and presentation of results: tabular, visual or even descriptive presentation of the results following a consistent logic. [17]

- Other aspects which might be relevant in conduct of the review: consultation phase in which end-users and practitioners can be involved (via focus group discussions, interviews or surveys) in order to identify alternative sources of information, to receive different perspectives on the data [28] and to improve the external validity of review results [30]. This phase is sometimes considered optional [16] and considered to be an essential part by others (e.g. [30]) in conducting a Scoping Review.

[…] as Review of Reviews

A Review of Reviews (also referred to as 'Overview of Reviews') is a type of review in which reviews and not individual studies are considered as primary evidence sources, requiring adjustments to the search, selection, extraction, quality appraisal, and analysis process [21, 24]. This review type is particularly useful if several reviews (with or without mixed results) already exist sharing a similar or the same research question and the quality of the individual reviews is unknown prior to conduct of the review. Methodological quality assessments can be carried out by means of using an appropriate appraisal tool for Systematic Reviews, AMSTAR 2 in particular. Statistical Meta-Analyses are rarely feasible. Usually this requires individual study results data of every primary study, which were part of any included review (see: [...] with Meta-Analysis). Otherwise, a Meta-analysis based on multiple data from one and the same study source (e.g. by pooling reviews with overlap in studies, not individual studies) would be based on biased weights. For Review of Reviews this should be carefully taken into consideration, especially when results are discussed and reported at the individual study level - a relatively time-consuming process for review authors [21].

Key differences compared to core elements that lay the foundation for Systematic Reviews

- Study selection: 'Reviews' as eligible evidence sources. [21, 24]

- Methods selection (e.g. as documented in the review protocol e.g. active record on PROSPERO): appropriate methods suitable to data of reviews used consistently throughout data extraction, quality assessment (e.g. AMSTAR 2), data processing and synthesis, in particular to avoid biased weighting of or through disproportionate coverage on individual studies from included reviews. [21]

[…] with Mixed-Methods-Approach

Reviews with a "Mixed-Methods-Approach" are used to combine quantitative and qualitative (primary study) data or to integrate quantitative and qualitative evidence in order to create a broader and deeper understanding, confirm or challenge study results and to ultimately answer the review question under consideration [17]. In principle, three levels of "Mixed-Method" review approaches are available: (i) the evidence sources included in the review follow either a quantitative or qualitative approach (as well as studies which provide data on both types), so that a combined synthesis can be conducted, (ii) the synthesis methods used in the review consider both quantitative (e.g. Meta-Analysis) and qualitative synthesis procedures (e.g. Meta-Synthesis, Meta-Ethnography) equally, (iii) the review uses two different modes of analysis: theory/hypothesis formation or theory/hypothesis testing. In general, a review to be rightfully self-labelled as using a Mixed-Methods-Approach should be reflected in the variety of quantitative and qualitative evidence sources included and in far-reaching adjustments of review methods, the presentation of results and/or the integration of results [22]. This narrow definition described here, which focuses on qualitative and quantitative research output identified by a systematic search process which is later analysed in the review, should not be used for reviews, for example, in which a quantitative synthesis is supplemented with qualitative data specifically collected for the review.

Key differences compared to review types without a Mixed-Methods-Approach

- Methods section of the review or review protocol (e.g. active record on PROSPERO or OSF, contractual document): additional information on the analysis plan provided, whether analyses (i) will be first performed separately from each other or in parallel according to the included qualitative and quantitative data and its structure and then integrated into a mixed methods synthesis ("segregated synthesis"), or (ii) a combined approach for qualitative and quantitative evidence sources will be used (e.g. by a subsequent data conversion into a comparable format) (assimilated data; "integrated synthesis"), or (iii) a sequential analysis based on each other ("contingent synthesis") will be conducted. [22]
- Study selection: evidence sources using a quantitative or qualitative approach are both eligible for inclusion (as well as studies which provide data on both types). [17, 22]
- Methods selection: appropriate methods suitable to both quantitative and qualitative data used consistently throughout data extraction, quality assessment, data processing (e.g. Bayesian conversion) and synthesis. [22]

[…] with Meta-Analysis

If included studies or evidence sources are sufficiently statistically and methodologically homogeneous, relevant data of primary studies can be often synthesised using a Meta-Analysis approach (e.g. visually presented as a forest plot). If applicable or necessary, subgroup and sensitivity analyses can be calculated additionally to determine the influence of effect estimates sharing a particular characteristic, e.g. high risk of bias, on a given summary effect estimate [1, 4, 17]. Results provided by using additional analytical methods such as the funnel plot can also provide further proof to support or invalidate statements on potential publication bias [4].

Key differences compared to review types without Meta-Analysis

- Quantitative synthesis: Meta-Analysis based on a justified choice of a particular method for calculating individual weights and visual presentation of results by using appropriate plots (e.g. forest plots, funnel plots). [1, 4, 17]
- Homo-/heterogeneity of studies: determined by using statistical methods/tests. [1, 4, 17]

References

1. Moher D, Liberati A, Tetzlaff J, Altman DG: **Preferred reporting items for systematic reviews and meta-analyses: the PRISMA statement**. PLoS medicine 2009, **6**(7):e1000097.
2. Shamseer L, Moher D, Clarke M, Ghersi D, Liberati A, Petticrew M, Shekelle P, Stewart LA: **Preferred reporting items for systematic review and meta-analysis protocols (PRISMA-P) 2015: elaboration and explanation**. BMJ 2015, **350**:g7647.
3. Tricco AC, Lillie E, Zarin W, O'Brien KK, Colquhoun H, Levac D, Moher D, Peters MDJ, Horsley T, Weeks L et al: **PRISMA Extension for Scoping Reviews (PRISMA-ScR): Checklist and Explanation**. Annals of Internal Medicine 2018, **169**(7):467-473.
4. Higgins JPT, Lasserson T, Chandler J, Tovey D, Churchill R: **Methodological Expectations of Cochrane Intervention Reviews** [https://community.cochrane.org/mecir-manual/; Accessed 24.07.2019]
5. Mulrow CD: **The Medical Review Article: State of the Science**. Annals of Internal Medicine 1987, **106**(3):485-488.
6. Oxman AD, Guyatt GH: **Validation of an index of the quality of review articles**. Journal of Clinical Epidemiology 1991, **44**(11):1271-1278.
7. Shea BJ, Reeves BC, Wells G, Thuku M, Hamel C, Moran J, Moher D, Tugwell P, Welch V, Kristjansson E et al: **AMSTAR 2: a critical appraisal tool for systematic reviews that include randomised or non-randomised studies of healthcare interventions, or both**. BMJ 2017, **358**:j4008.
8. Health Evidence: **Healthevidence.org: Quality Assessment Tool - Review Articles** [https://www.healthevidence.org/documents/our-appraisal-tools/QA_Tool&Dictionary_10Nov16.pdf; Accessed 24.07.2019]
9. Whiting P, Savović J, Higgins JPT, Caldwell DM, Reeves BC, Shea B, Davies P, Kleijnen J, Churchill R, group R: **ROBIS: A new tool to assess risk of bias in systematic reviews was developed**. Journal of Clinical Epidemiology 2016, **69**:225-234.
10. Critical Appraisal Skills Programme: **CASP Checklist: 10 questions to help you make sense of a Systematic Review** [https://casp-uk.net/wp-content/uploads/2018/01/CASP-Systematic-Review-Checklist_2018.pdf; Accessed 24.07.2019]

11. Knowledge Translation Program: **Systematic review (of therapy) worksheet** [https://ebm-tools.knowledgetranslation.net/themes/blue/files/uploads/sr-worksheet.doc; Accessed 24.07.2019]
12. Scottish Intercollegiate Guidelines Network: **Critical appraisal notes and checklists - Methodology Checklist 1: Systematic Reviews and Meta-analyses** [https://www.sign.ac.uk/checklists-and-notes.html; Accessed 24.07.2019]
13. National Heart, Lung, and Blood Institute: **Quality Assessment of Systematic Reviews and Meta-Analyses** [https://www.nhlbi.nih.gov/health-topics/study-quality-assessment-tools; Accessed 24.07.2019]
14. Public Health Agency of Canada: **Infection Prevention and Control Guidelines: Critical Appraisal Tool Kit** [http://publications.gc.ca/collections/collection_2014/aspc-phac/HP40-119-2014-eng.pdf; Accessed 24.07.2019]
15. GKV-Bündnis für Gesundheit: **Das GKV-Bündnis für Gesundheit** [https://www.gkv-buendnis.de/; Accessed 14.05.2020]
16. Higgins JPT, Thomas J, Chandler J, Cumpston M, Li T, Page MJ, Welch VA: **Cochrane Handbook for Systematic Reviews of Interventions**, 2nd edn. Chichester (UK): John Wiley & Sons; 2019.
17. Aromataris E, Munn Z: **Joanna Briggs Institute Reviewer's Manual** [https://reviewersmanual.joannabriggs.org/ Accessed 24.07.2019]
18. Peters MDJ, Godfrey CM, Khalil H, McInerney P, Parker D, Soares CB: **Guidance for conducting systematic scoping reviews**. International Journal of Evidence-Based Healthcare 2015, **13**(3):141-146.
19. Tricco AC, Langlois EV, Straus S: **Rapid reviews to strengthen health policy and systems: a practical guide** [https://apps.who.int/iris/bitstream/handle/10665/258698/9789241512763-eng.pdf; Accessed 29.07.2019]
20. National Institute for Health Care Excellence: **Developing NICE Guidelines: The Manual** [https://www.nice.org.uk/process/pmg20/chapter/introduction-and-overview; Accessed 24.07.2019]
21. Smith V, Devane D, Begley CM, Clarke M: **Methodology in conducting a systematic review of systematic reviews of healthcare interventions**. BMC Medical Research Methodology 2011, **11**(1):15.
22. Pearson A, White H, Bath-Hextall F, Salmond S, Apostolo J, Kirkpatrick P: **A mixed-methods approach to systematic reviews**. International Journal of Evidence-Based Healthcare 2015, **13**(3):121-131.
23. Munn Z, Peters MDJ, Stern C, Tufanaru C, McArthur A, Aromataris E: **Systematic review or scoping review? Guidance for authors when choosing between a systematic or scoping review approach**. BMC Medical Research Methodology 2018, **18**(1):143.
24. Grant MJ, Booth A: **A typology of reviews: an analysis of 14 review types and associated methodologies**. Health Information & Libraries Journal 2009, **26**(2):91-108.
25. Hartling L, Guise JM, Kato E: **EPC Methods: An Exploration of Methods and Context for the Production of Rapid Reviews** [https://www.ncbi.nlm.nih.gov/books/NBK274092/pdf/Bookshelf_NBK274092.pdf; Accessed 29.07.2019]
26. Tricco AC, Lillie E, Zarin W, O'Brien K, Colquhoun H, Kastner M, Levac D, Ng C, Sharpe JP, Wilson K et al: **A scoping review on the conduct and reporting of scoping reviews**. BMC Medical Research Methodology 2016, **16**:15.
27. Harker J, Kleijnen J: **What is a rapid review? A methodological exploration of rapid reviews in Health Technology Assessments**. International Journal of Evidence-Based Healthcare 2012, **10**(4):397-410.
28. Arksey H, O'Malley L: **Scoping studies: towards a methodological framework**. International Journal of Social Research Methodology 2005, **8**(1):19-32.

29. Pham MT, Rajic A, Greig JD, Sargeant JM, Papadopoulos A, McEwen SA: **A scoping review of scoping reviews: advancing the approach and enhancing the consistency**. Research Synthesis Methods 2014, **5**(4):371-385.
30. Levac D, Colquhoun H, O'Brien KK: **Scoping studies: advancing the methodology**. Implementation Science 2010, **5**:69.
31. Khangura S, Polisena J, Clifford TJ, Farrah K, Kamel C: **Rapid review: an emerging approach to evidence synthesis in health technology assessment**. International Journal of Technology Assessment in Health Care 2014, **30**(1):20-27.
32. Shea BJ, Grimshaw JM, Wells GA, Boers M, Andersson N, Hamel C, Porter AC, Tugwell P, Moher D, Bouter LM: **Development of AMSTAR: a measurement tool to assess the methodological quality of systematic reviews**. BMC Medical Research Methodology 2007, **7**:10.
33. Task Force on Systematic Review and Guidelines: **Assessing the quality and applicability of systematic reviews (AQASR)** [https://ktdrr.org/ktlibrary/articles_pubs/ncddrwork/aqasr/; Accessed 24.07.2019]
34. European Food Safety Authority: **Tools for critically appraising different study designs, systematic review and literature searches** [https://doi.org/10.2903/sp.efsa.2015.EN-836; Accessed 24.07.2019]
35. The Joanna Briggs Institute: **Critical Appraisal tools for use in JBI Systematic Reviews: Checklist for Systematic Reviews and Research Syntheses** [http://joannabriggs.org/research/critical-appraisal-tools.html; Accessed 24.07.2019]
36. National Institute for Health Care Excellence: **The social care guidance manual - Appendix B Methodology checklist: systematic reviews and meta-analyses** [https://www.nice.org.uk/process/pmg10/chapter/appendix-b-methodology-checklist-systematic-reviews-and-meta-analyses; Accessed 24.07.2019]
37. Specialist Unit for Review Evidence: **Questions to assist with the critical appraisal of a systematic review** [https://www.cardiff.ac.uk/__data/assets/pdf_file/0007/1142962/SURE-CA-form-for-SR_2018.pdf; Accessed 24.07.2019]
38. Goudet SM, Bogin BA, Madise NJ, Griffiths PL: **Nutritional interventions for preventing stunting in children (birth to 59 months) living in urban slums in low- and middle-income countries (LMIC)**. Cochrane Database of Systematic Reviews 2019(6).
39. Deeks JJ, Higgins JP, Altman DG: **Analysing data and undertaking meta-analyses**. In: Cochrane Handbook for Systematic Reviews of Interventions. 2nd edn. Edited by Higgins JP, Thomas J, Chandler J, Cumpston M, Li T, Page MJ, Welch VA. Hoboken, NJ: The Cochrane Collaboration and John Wiley & Sons Ltd; 2019: 241-284.
40. McKenzie JE, Brennan SE: **Synthesizing and presenting findings using other methods**. In: Cochrane Handbook for Systematic Reviews of Interventions. 2nd edn. Edited by Higgins JP, Thomas J, Chandler J, Cumpston M, Li T, Page MJ, Welch VA. Hoboken, NJ: The Cochrane Collaboration and John Wiley & Sons Ltd; 2019: 321-347.
41. McKenzie JE, Brennan SE, Ryan RE, Thomson HJ, Johnston RV: **Summarizing study characteristics and preparing for synthesis**. In: Cochrane Handbook for Systematic Reviews of Interventions. 2nd edn. Edited by Higgins JP, Thomas J, Chandler J, Cumpston M, Li T, Page MJ, Welch VA. Hoboken, NJ: The Cochrane Collaboration and John Wiley & Sons Ltd; 2019: 229-240.
42. Schünemann HJ, Vist GE, Higgins JP, Santesso N, Deeks JJ, Glasziou P, Akl EA, Guyatt GH: **Interpreting results and drawing conclusions**. In: Cochrane Handbook for Systematic Reviews of Interventions. 2nd edn. Edited by Higgins JP, Thomas J, Chandler J, Cumpston M, Li T, Page MJ, Welch VA. Hoboken, NJ: The Cochrane Collaboration and John Wiley & Sons Ltd; 2019: 403-431.
43. Effective Public Health Practice Project: **Quality Assessment Tool For Quantitative Studies** [https://merst.ca/wp-content/uploads/2018/02/quality-assessment-tool_2010.pdf; Accessed 24.07.2019]

44. Munthe-Kaas HM, Glenton C, Booth A, Noyes J, Lewin S: **Systematic mapping of existing tools to appraise methodological strengths and limitations of qualitative research: first stage in the development of the CAMELOT tool**. BMC Medical Research Methodology 2019, **19**(1):113.
45. Tricco AC, Antony J, Zarin W, Strifler L, Ghassemi M, Ivory J, Perrier L, Hutton B, Moher D, Straus SE: **A scoping review of rapid review methods**. BMC Medicine 2015, **13**:224.
46. Nussbaumer-Streit B, Klerings I, Wagner G, Heise TL, Dobrescu AI, Armijo-Olivo S, Stratil JM, Persad E, Lhachimi SK, Van Noord MG et al: **Abbreviated literature searches were viable alternatives to comprehensive searches: a meta-epidemiological study**. Journal of Clinical Epidemiology 2018, **102**:1-11.
