# Supplementary material for: CAT HPPR: a critical appraisal tool to assess the quality of systematic, rapid, and scoping reviews investigating interventions in health promotion and prevention
Source: BMC Med Res Methodol. 2022 Dec 26;22:334. doi: 10.1186/s12874-022-01821-4 (PMC9791771; doi:10.1186/s12874-022-01821-4)
Supplement: Supplementary file 2 — Additional file 2. [file 12874_2022_1821_MOESM2_ESM.pdf]

Supplementary file 1. Search documentation and item retrieval

1. SEARCH DOCUMENTATION: CATs 2
MEDLINE via Ovid 2
REPOSITORY 2
WEBSITES 3
REFERENCE LIST OF KEY DOCUMENT 3
GKV-BÜNDNIS (HANDSEARCH) 4
1.1 FLOW CHART FOR IDENTIFYING CATs 5
2. SEARCH DOCUMENTATION: REPORTING GUIDELINES AND GUIDANCE DOCUMENTS. 5
MEDLINE via Ovid 5
REPOSITORY 6
WEBSITES 6
GKV-BÜNDNIS (HANDSEARCH) 7
2.1 FLOW CHART FOR SELECTING GUIDELINES AND GUIDANCE DOCUMENTS 8
3. COMPARSION HEALTHEVIDENCE.ORG VERSUS OTHER INCLUDED CATS 9
4. SHORT-LIST OF 46 ITEMS BASED ON ALL INCLUDED CATs 10

1. SEARCH DOCUMENTATION: CATs

MEDLINE via Ovid

[24/07/2019; 220 hits]

1. "Systematic Reviews as Topic"/
2. critical appraisal tool?.ab,kf,ti.
3. CAT?.ab,kf,ti.
4. (quality assessment adj2 tool?).ab,kf,ti.
5. (quality appraisal adj2 tool?).ab,kf,ti.
6. (methodological quality adj2 tool?).ab,kf,ti.
7. 1 and (2 or 3 or 4 or 5 or 6)
8. (critical appraisal tool? adj4 (review or reviews)).ab,kf,ti.
9. (CAT? adj4 (review or reviews)).ab,kf,ti.
10. (quality assessment tool? adj4 (review or reviews)).ab,kf,ti.
11. (quality appraisal adj2 tool?) adj4 (review or reviews).ab,kf,ti.
12. (methodological quality adj2 tool?) adj4 (review or reviews).ab,kf,ti.
13. or/8-12
14. 7 or 13
15. exp animals/ not humans.sh.
16. 14 not 15

Table 1 MEDLINE search via Ovid (CATs)

REPOSITORY

National Collaborating Centre for Methods and Tools (https://www.nccmt.ca/knowledge-repositories/search; search terms “appraisal” and “assessment”; [24/07/2019])

Main report of CAT retrieved (multiple records (article on development, validation etc.) of one CAT were limited to the main report throughout this document):

- **CASP:** CASP Checklist: 10 questions to help you make sense of a Systematic Review https://casp-uk.net/wp-content/uploads/2018/01/CASP-Systematic-Review-Checklist_2018.pdf Accessed 24 July 2019
- **JBI CAT:** Critical Appraisal tools for use in JBI Systematic Reviews: Checklist for Systematic Reviews and Research Syntheses http://joannabriggs.org/research/critical-appraisal-tools.html Accessed 24 July 2019
- **AMSTAR 2:** Shea BJ, Reeves BC, Wells G, Thuku M, Hamel C, Moran J, Moher D, Tugwell P, Welch V, Kristjansson E et al: AMSTAR 2: a critical appraisal tool for systematic reviews that include randomised or non-randomised studies of healthcare interventions, or both. BMJ 2017, 358:j4008.
- **ROBIS:** Whiting P, Savović J, Higgins JPT, Caldwell DM, Reeves BC, Shea B, Davies P, Kleijnen J, Churchill R, group R: ROBIS: A new tool to assess risk of bias in systematic reviews was developed. J Clin Epidemiol 2016, 69:225-234.
- **Healthevidence.org:** Quality Assessment Tool - Review Articles https://www.healthevidence.org/documents/our-appraisal-tools/QA_Tool&Dictionary_10Nov16.pdf Accessed 24 July 2019
- **MetaQAT:** Meta-tool for quality appraisal of public health evidence - PHO MetaQAT 1.0 https://www.publichealthontario.ca/en/ServicesAndTools/CriticalAppraisalTool/PHO_MetaQAT_2015.pdf Accessed 24 July 2019
- **PHAC CATK:** Infection Prevention and Control Guidelines: Critical Appraisal Tool Kit http://publications.gc.ca/collections/collection_2014/aspc-phac/HP40-119-2014-eng.pdf Accessed 24 July 2019

WEBSITES

Joanna Briggs Institute (https://jbi.global/critical-appraisal-tools; [24/07/2019])

Main report of CAT retrieved:

- **JBI CAT:** Critical Appraisal tools for use in JBI Systematic Reviews: Checklist for Systematic Reviews and Research Syntheses http://joannabriggs.org/research/critical-appraisal-tools.html Accessed 24 July 2019

NICE (https://www.nice.org.uk/process/pmg10/chapter/appendix-b-methodology-checklist-systematic-reviews-and-meta-analyses; [24/07/2019])

Main report of CAT retrieved:

- **NICE SCG:** The social care guidance manual - Appendix B Methodology checklist: systematic reviews and meta-analyses https://www.nice.org.uk/process/pmg10/chapter/appendix-b-methodology-checklist-systematic-reviews-and-meta-analyses Accessed 24 July 2019

REFERENCE LIST OF KEY DOCUMENT

Cochrane Deutschland, Arbeitsgemeinschaft der Wissenschaftlichen Medizinischen Fachgesellschaften – Institut für Medizinisches Wissensmanagement. „Bewertung von systematischen Übersichtsarbeiten: ein Manual für die Leitlinienerstellung“ [Assessment of systematic reviews: a manual for guideline development]. [http://www.cochrane.de/de/review-bewertung-manual]; [http://www.awmf.org/leitlinien/awmf-regelwerk/ll-entwicklung.html]

Main report of CAT retrieved:

- **ROBIS:** Whiting P, Savović J, Higgins JPT, Caldwell DM, Reeves BC, Shea B, Davies P, Kleijnen J, Churchill R, group R: ROBIS: A new tool to assess risk of bias in systematic reviews was developed. J Clin Epidemiol 2016, 69:225-234.
- **Healthevidence.org:** Quality Assessment Tool - Review Articles https://www.healthevidence.org/documents/our-appraisal-tools/QA_Tool&Dictionary_10Nov16.pdf Accessed 24 July 2019
- **DART:** Diekemper RL, Ireland BK, Merz LR: Development of the Documentation and Appraisal Review Tool for systematic reviews. World J Meta-Anal 2015, 3(3):142-150.
- **NIH NHLBI:** Quality Assessment of Systematic Reviews and Meta-Analyses https://www.nhlbi.nih.gov/health-topics/study-quality-assessment-tools Accessed 24 July 2019
- **CEBMA:** Critical Appraisal of a Meta-analysis or Systematic Review https://www.cebma.org/wp-content/uploads/Critical-Appraisal-Questions-for-a-SR-or-MA-july-2014.pdf Accessed 24 July 2019
- **SURE:** Questions to assist with the critical appraisal of a systematic review https://www.cardiff.ac.uk/__data/assets/pdf_file/0007/1142962/SURE-CA-form-for-SR_2018.pdf Accessed 24 July 2019
- **CASP:** CASP Checklist: 10 questions to help you make sense of a Systematic Review https://casp-uk.net/wp-content/uploads/2018/01/CASP-Systematic-Review-Checklist_2018.pdf Accessed 24 July 2019
- **ANDEAL:** Evidence Analysis Manual - Appendix 10: Quality Criteria Checklist: Review Article https://www.andeal.org/vault/2440/web/files/2016_April_EA_Manual.pdf Accessed 24 July 2019

- **R-AMSTAR:** Kung J, Chiappelli F, Cajulis OO, Avezova R, Kossan G, Chew L, Maida CA: From Systematic Reviews to Clinical Recommendations for Evidence-Based Health Care: Validation of Revised Assessment of Multiple Systematic Reviews (R-AMSTAR) for Grading of Clinical Relevance. Open Dent J 2010, 4:84-91.
- **AMSTAR:** Shea BJ, Grimshaw JM, Wells GA, Boers M, Andersson N, Hamel C, Porter AC, Tugwell P, Moher D, Bouter LM: Development of AMSTAR: a measurement tool to assess the methodological quality of systematic reviews. BMC Med Res Methodol 2007, 7:10.
- **OQAQ Oxman et al:** Oxman AD, Guyatt GH: Validation of an index of the quality of review articles. J Clin Epidemiol 1991, 44(11):1271-1278.
- **CEBM:** Critical Appraisal Worksheets: Systematic Reviews https://www.cebm.net/systematic-review-4/ Accessed 24 July 2019
- **JBI CAT:** Critical Appraisal tools for use in JBI Systematic Reviews: Checklist for Systematic Reviews and Research Syntheses http://joannabriggs.org/research/critical-appraisal-tools.html Accessed 24 July 2019
- **DUKE:** Assessing the Credibility of the Systematic Review Process https://guides.mclibrary.duke.edu/ld.php?content_id=27688601 Accessed 24 July 2019
- **GP UG CAC:** Critical Appraisal Checklist for a Systematic Review https://www.gla.ac.uk/media/media_64047_en.pdf Accessed 24 July 2019
- **KTP:** Systematic review (of therapy) worksheet https://ebm-tools.knowledgetranslation.net/themes/blue/files/uploads/sr-worksheet.doc Accessed 24 July 2019
- **SIGN:** Critical appraisal notes and checklists - Methodology Checklist 1: Systematic Reviews and Meta-analyses https://www.sign.ac.uk/checklists-and-notes.html Accessed 24 July 2019
- **BET:** Reviews and Meta-Analyses Checklist https://bestbets.org/ca/pdf/review.pdf Accessed 24 July 2019

GKV-BÜNDNIS (HANDSEARCH)

References of CATs provided by the GKV-Bündnis or already known by the author team not identified by search approaches previously described [31/07/2019]

Main report of CAT retrieved:

- **AQASAR:** Assessing the quality and applicability of systematic reviews (AQASR) https://ktdrr.org/ktlibrary/articles_pubs/ncddrwork/aqasr/ Accessed 24 July 2019
- **EFSA CAT:** Tools for critically appraising different study designs, systematic review and literature searches https://doi.org/10.2903/sp.efsa.2015.EN-836 Accessed 24 July 2019
- **Glenny et al:** Glenny AM, Esposito M, Coulthard P, Worthington HV: The assessment of systematic reviews in dentistry. Eur J Oral Sci 2003, 111(2):85-92.
- **Mulrow et al:** Mulrow CD: The Medical Review Article: State of the Science. Ann Intern Med 1987, 106(3):485-488.
- **Sacks et al:** Sacks HS, Berrier J, Reitman D, Ancona-Berk VA, Chalmers TC: Meta-Analyses of Randomized Controlled Trials. N Engl J Med 1987, 316(8):450-455.

1.1 FLOW CHART FOR IDENTIFYING CATs

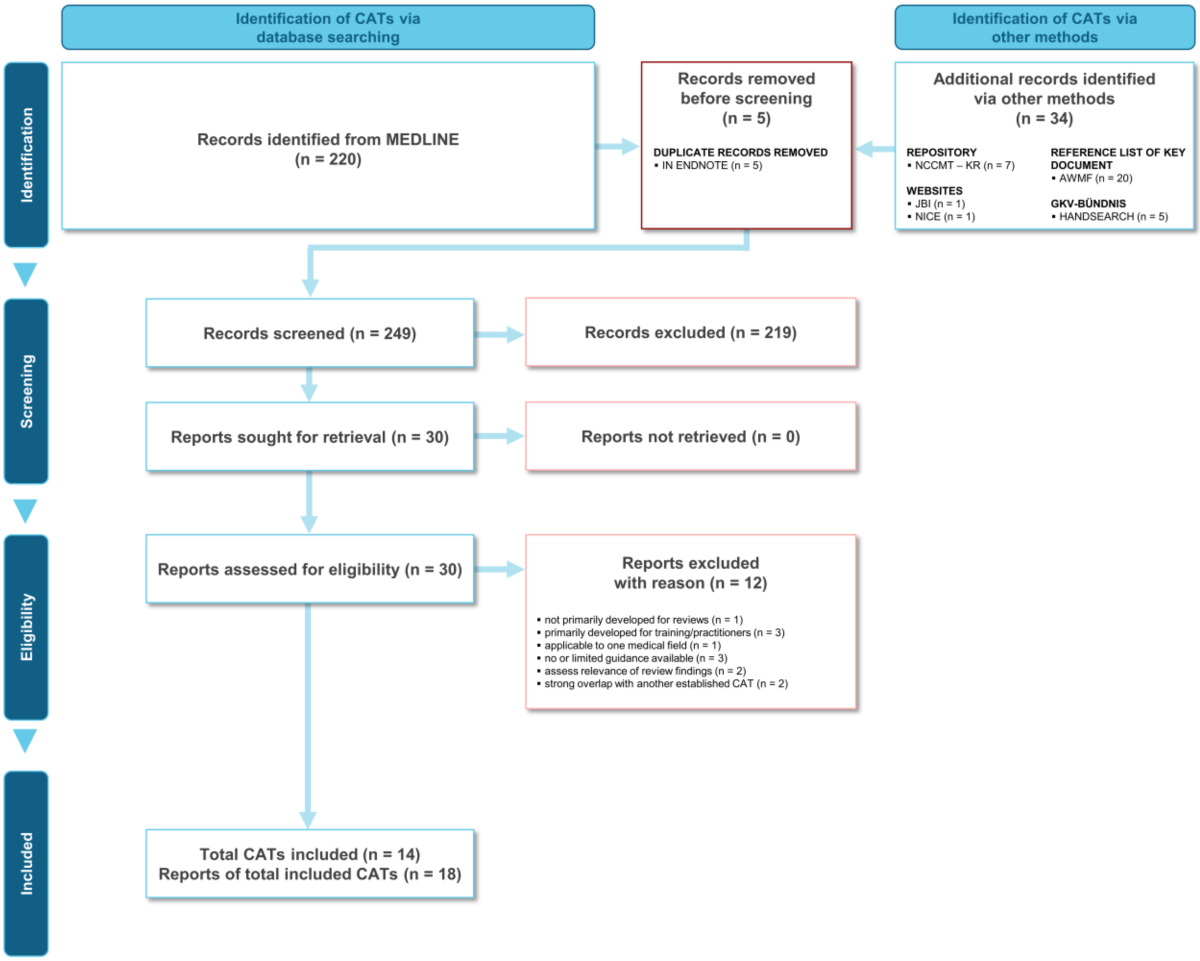

Figure 1 Flow chart for identifying CATs

Notes after completing the CAT HPPR development process [24/07/2020]:
inclusion of “risk of bias” as a search concept and exclusion of, or different use of “tool” could potentially lead to improved information retrieval with regard to database searches in future updates (see Table 1; major downside to consider: high proportion of irrelevant search results). However, many CATs, especially those published as grey literature, were successfully retrieved by searching other resources (e.g. see CATs identified by searching NCCMT repository or key document). That is why, searching reference lists of other key documents retrieved by database searches should be considered for future updates of the tool or reviews on the same topic (e.g. articles on the applicability of different CATs for various review products).

2. SEARCH DOCUMENTATION: REPORTING GUIDELINES AND GUIDANCE DOCUMENTS

MEDLINE via Ovid

[24/07/2019; 49 hits]

1. "Systematic Reviews as Topic"/<br>2. (review or reviews).ti.<br>3. (reporting adj2 guideline).ti.<br>4. (reporting adj2 preferred).ti.<br>5. (reporting adj2 conduct*).ti.<br>6. (guidance adj2 conduct*).ti.<br>7. (guideline adj2 conduct*).ti.

8. or/1-2
9. or/3-7
10. 8 and 9
11. exp animals/ not humans.sh.
12. 10 not 11

Table 2 MEDLINE search via Ovid (guidelines)

REPOSITORY

EQUATOR Network (https://www.equator-network.org/reporting-guidelines/; Study type: ‘Systematic reviews/Meta-analyses/Reviews/HTA/Overviews’; selection limited to ‘Key reporting guidelines’ [24/07/2019])

Main report of guideline retrieved:

- **PRISMA:** Moher D, Liberati A, Tetzlaff J, Altman DG: Preferred reporting items for systematic reviews and meta-analyses: the PRISMA statement. PLoS Med 2009, 6(7):e1000097.
- **PRISMA-ScR:** Tricco AC, Lillie E, Zarin W, O'Brien KK, Colquhoun H, Levac D, Moher D, Peters MDJ, Horsley T, Weeks L et al: PRISMA Extension for Scoping Reviews (PRISMA-ScR): Checklist and Explanation. Ann Intern Med 2018, 169(7):467-473.
- **PRISMA-P:** Shamseer L, Moher D, Clarke M, Ghersi D, Liberati A, Petticrew M, Shekelle P, Stewart LA: Preferred reporting items for systematic review and meta-analysis protocols (PRISMA-P) 2015: elaboration and explanation. BMJ 2015, 350:g7647.

WEBSITES

Cochrane (https://community.cochrane.org/mecir-manual; https://training.cochrane.org/handbook [24/07/2019])

Main report of guidance retrieved:

- **MECIR:** Methodological Expectations of Cochrane Intervention Reviews https://community.cochrane.org/mecir-manual/ Accessed 24 July 2019
- **Cochrane Handbook:** Cochrane Handbook for Systematic Reviews of Interventions Version 5.1.0 [updated March 2011] https://training.cochrane.org/handbook Accessed 24 July 2019

JBI (https://reviewersmanual.joannabriggs.org/ [24/07/2019]; new link as of [31/10/2022]: https://jbi-global-wiki.refined.site/space/MANUAL)

Main report of guidance retrieved:

- **JBI RM:** Joanna Briggs Institute Reviewer's Manual https://reviewersmanual.joannabriggs.org/ Accessed 24 July 2019

NICE (https://www.nice.org.uk/ [24/07/2019])

Main report of guidance retrieved:

- **NICE:** Developing NICE Guidelines: The Manual https://www.nice.org.uk/process/pmg20/chapter/introduction-and-overview Accessed 24 July 2019

GKV-BÜNDNIS (HANDSEARCH)

References of guidance documents provided by the GKV-Bündnis or already known by the author team not identified by other search approaches previously described [31/07/2019]

Main report of guidance retrieved:

- Grant MJ, Booth A: A typology of reviews: an analysis of 14 review types and associated methodologies. Health Info Libr J 2009, 26(2):91-108.
- Arksey H, O'Malley L: Scoping studies: towards a methodological framework. Int J Soc Res Methodol 2005, 8(1):19-32.
- Harker J, Kleijnen J: What is a rapid review? A methodological exploration of rapid reviews in Health Technology Assessments. Int J Evid Based Healthc 2012, 10(4):397-410.
- EPC Methods: An Exploration of Methods and Context for the Production of Rapid Reviews https://www.ncbi.nlm.nih.gov/books/NBK274092/pdf/Bookshelf_NBK274092.pdf Accessed 24 July 2019
- Khangura S, Polisena J, Clifford TJ, Farrah K, Kamel C: Rapid review: an emerging approach to evidence synthesis in health technology assessment. Int J Technol Assess Health Care 2014, 30(1):20-27.
- Levac D, Colquhoun H, O'Brien KK: Scoping studies: advancing the methodology. Implement Sci 2010, 5:69.
- Munn Z, Peters MDJ, Stern C, Tufanaru C, McArthur A, Aromataris E: Systematic review or scoping review? Guidance for authors when choosing between a systematic or scoping review approach. BMC Med Res Methodol 2018, 18(1):143.
- Pearson A, White H, Bath-Hextall F, Salmond S, Apostolo J, Kirkpatrick P: A mixed-methods approach to systematic reviews. Int J Evid Based Healthc 2015, 13(3):121-131.
- Pham MT, Rajic A, Greig JD, Sargeant JM, Papadopoulos A, McEwen SA: A scoping review of scoping reviews: advancing the approach and enhancing the consistency. Res Synth Methods 2014, 5(4):371-385.
- Smith V, Devane D, Begley CM, Clarke M: Methodology in conducting a systematic review of systematic reviews of healthcare interventions. BMC Med Res Methodol 2011, 11(1):15.
- Rapid reviews to strengthen health policy and systems: a practical guide https://apps.who.int/iris/bitstream/handle/10665/258698/9789241512763-eng.pdf Accessed 29 July 2019

2.1 FLOW CHART FOR SELECTING GUIDELINES AND GUIDANCE DOCUMENTS

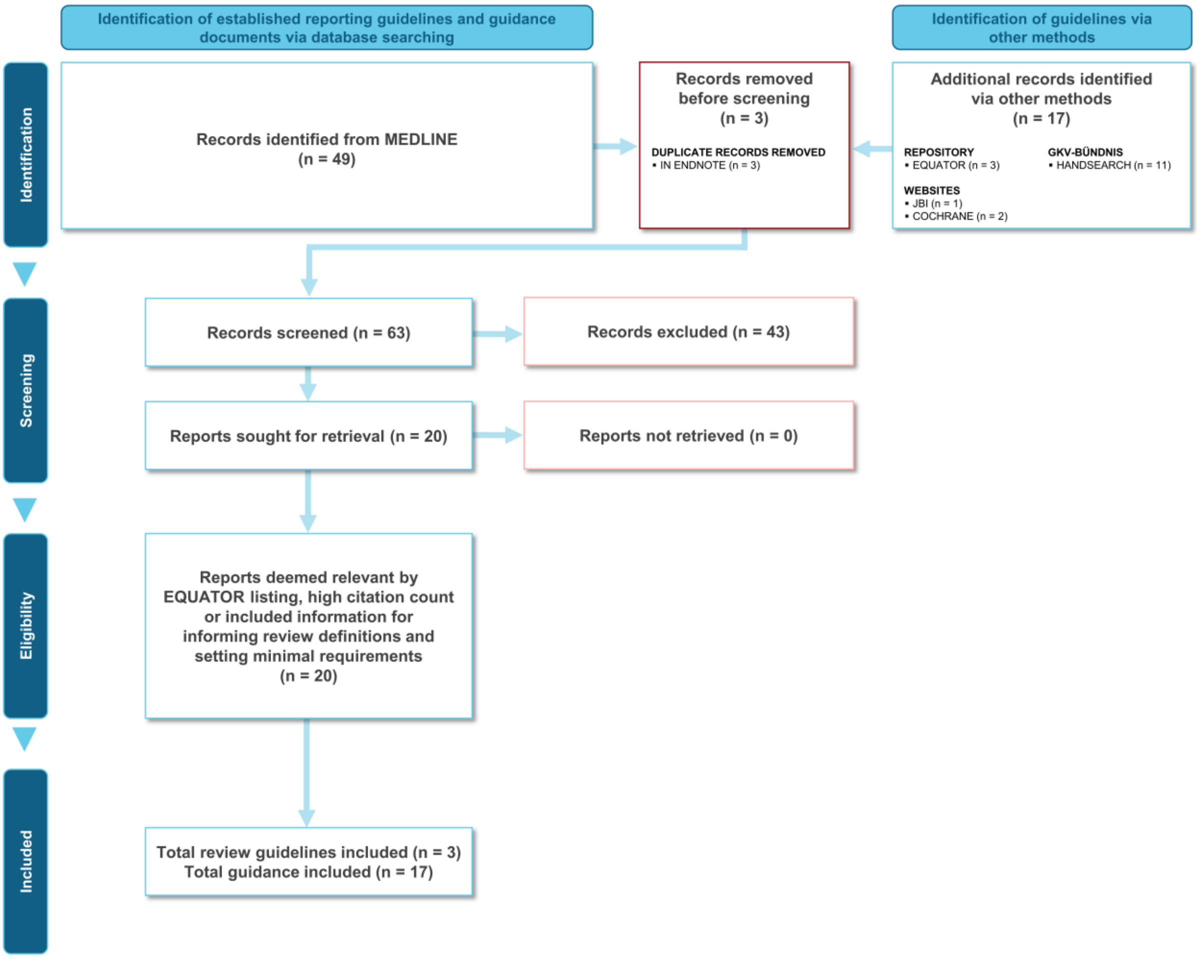

Figure 2 Abbreviated flow chart to summarise retrieval of reporting guidelines and guidance documents

Notes after completing the CAT HPPR development process [24/07/2020]:
our search and screening approach for identifying review guidelines and guidance documents was by design pragmatic and abbreviated compared to searches found in systematic reviews. We did not exclude reports in the full-text screening stage as the selection process of records was mainly driven by other methods than database searches (i.e. repository, websites, pre-identified documents). For reporting guidelines, journals and researchers commonly refer to PRISMA and PRISMA-extension as gold-standard. The same is true for methodological guidance of JBI and Cochrane for conducting a variety of different review products (e.g. (Cochrane) systematic reviews, scoping reviews). All other guidance documents identified by our search were, therefore, mainly used to fill gaps in defining review types (e.g. RR) and complementary approaches not well-described by PRISMA reporting guidelines or other guidance published by Cochrane or JBI at the time the search was conducted.

3. COMPARSION HEALTHEVIDENCE.ORG VERSUS OTHER INCLUDED CATS

main theme of healthevidence.org item	AMSTAR 2	AMSTAR	AQASR	CASP	EFSA CAT	JBI	NICE SCG	NIH NHLBI	OQAQ	PHAC	ROBIS	SIGN	SURE
Q1. focused PICO question													
Q2. appropriate inclusion criteria defined													
Q3. comprehensive search strategy (databases)													
Q4. years covered; search strategy													
Q5. evidence level of included primary studies reported													
Q6. quality assessment of included studies													
Q7. transparent reporting of review results (quality assessment by two authors)													
Q8. synthesis of data appropriate													
Q9. appropriate methods for data synthesis													
Q10. data support author's conclusions													

Table 3 Data extraction table to compare items of healthevidence.org with included items of other CATs

This abbreviated table in its original format served as a first comparison of items from healthevidence.org with those reported in other CATs during early stages of tool development (please note: all original comments (e.g. items of CATs) were removed due to space restrictions). In a second step (data not shown; see Availability of data and materials), items which were not part of healthevidence.org, but included in other CATs were presented to the project team to review, and a short-list of 46 items was created. Items were then either directly included as new criteria (see Table 1 of main article) or integrated into other criteria, while others were excluded by stating the reason for exclusion (see main article).

4. SHORT-LIST OF 46 ITEMS BASED ON ALL INCLUDED CATs

Q#	review part or problem addressed	original item description	source [ref. main article]
Q1	review question	"Did the authors have a clearly focused question [population, intervention (strategy), and outcome(s)]?"	Q1: healthevidence.org [15]
Q2	study selection	"Were appropriate inclusion criteria used to select primary studies?"	Q2: healthevidence.org [15]
Q3	search strategy	"Did the authors describe a search strategy that was comprehensive?"	Q3: healthevidence.org [15]
Q4	search strategy	"Did search strategy cover an adequate number of years?"	Q4: healthevidence.org [15]
Q5	level of evidence	"Did the authors describe the level of evidence in the primary studies included in the review?"	Q5: healthevidence.org [15]
Q6	quality assessment	"Did the review assess the methodological quality of the primary studies, including: Research design, Study sample, Participation rates, Sources of bias (confounders, respondent bias), Data collection (measurement of independent/dependent variables), Follow-up/attrition rates, Data analysis"	Q6: healthevidence.org [15]
Q7	quality assessment	"Are the results of the review transparent?"	Q7: healthevidence.org [15]
Q8	heterogeneity	"Was it appropriate to combine the findings of results across studies?"	Q8: healthevidence.org [15]
Q9	synthesis	"Were appropriate methods used for combining or comparing results across studies?"	Q9: healthevidence.org [15]
Q10	discussion of review findings/results	"Do the data support the author's interpretation?"	Q10: healthevidence.org [15]
Q11	general methods	"Did the report of the review contain an explicit statement that the review methods were established prior to conduct of the review and did the report justify any significant deviations from the protocol?"	item 2: AMSTAR 2 [16]
Q12	eligibility criteria	"Did the review authors explain their selection of the study designs for inclusion in the review?"	item 3: AMSTAR 2 [16]
Q13	study selection	"Did the review authors perform study selection in duplicate?"	item 5: AMSTAR 2 [16]
Q14	data extraction	"Did the review authors perform data extraction in duplicate?"	item 6: AMSTAR 2 [16]
Q15	study selection	"Did the review authors provide a list of excluded studies and justify the exclusions?"	item 7: AMSTAR 2 [16]
Q16	study selection	"Did the review authors describe the included studies in adequate detail?"	item 8: AMSTAR 2 [16]
Q17	conflict of interest	"Did the review authors report on the sources of funding for the studies included in the review?"	item 10: AMSTAR 2 [16]
Q18	discussion of review findings/results	"Did the review authors account for RoB in individual studies when interpreting/ discussing the results of the review?"	item 13: AMSTAR 2 [16]

Q19	heterogeneity	"Did the review authors provide a satisfactory explanation for, and discussion of, any heterogeneity observed in the results of the review?"	item 14: AMSTAR 2 [16]
Q20	publication bias	"If they performed quantitative synthesis did the review authors carry out an adequate investigation of publication bias (small study bias) and discuss its likely impact on the results of the review?"	item 15: AMSTAR 2 [16]
Q21	conflict of interest	"Did the review authors report any potential sources of conflict of interest, including any funding they received for conducting the review?"	item 16: AMSTAR 2 [16]
Q22	study selection	"Was the status of publication (i.e. grey literature) used as an inclusion criterion?"	item 4: AMSTAR [35]
Q23	rationale	"Is there a rationale for the review? Is the clinical/scientific background for the review discussed, the guiding problem defined?"	RQ2: AQASR [37]
Q24	rationale	"Do the authors refer to systematic reviews in this area done previously? Do they justify the need for a new review?"	RQ3: AQASR [37]
Q25	adverse effects	"Are (potential) harms described/defined?"	RQ5: AQASR [37]
Q26	study selection	"Is nature and training of abstract reviewers specified?"	SC2: AQASR [37]
Q27	study selection	"Is there a clear description or flow diagram describing the disposition of abstracts and papers through the various steps in the process of identifying the relevant evidence (abstracts read > full papers read > full papers extracted, etc.?)"	SC7: AQASR [37]
Q28	quality assessment	"Was the instrument for assessing study quality identified and presented? Was the choice of review instrument justified?"	MQ2: AQASR [37]
Q29	quality assessment	"Is nature and training of study quality scorers/reviewers specified?"	MQ5: AQASR [37]
Q30	data extraction	Is an extracting form and syllabus described? If so, is pilot testing of the form/ syllabus described?	DA1: AQASR [37]
Q31	data extraction	"Is the nature and training of the data extractors specified?"	DA4: AQASR [37]
Q32	discussion of review findings/results	"Are study limitations discussed (e.g. search limitations, the effects of publication and other biases, strength of studies, decisions on synthesis)?"	DI1: AQASR [37]
Q33	discussion of review findings/results	"If there were earlier systematic reviews in this area: Do the authors discuss similarity or differences in findings, and try to explain differences?"	DI6: AQASR [37]
Q34	discussion of review findings/results	"Were directions for future research proposed?"	DI7: AQASR [37]
Q35	discussion of review findings/results	"Overall assessment of external validity – Are the results externally valid (i.e. generalisable to the whole source population)? Consider participants, interventions, settings, comparisons and outcomes."	item 7: NICE SCG [30]

Q36	synthesis	"Was the number of studies that contributed to the decision regarding a clear effect sufficient (four or more)?"	item retrieved from text; no original item; description of Results c): PHAC [31]
Q37	discussion of review findings/results	"Were the results clearly described and interpreted in a meaningful way?"	item 6: PHAC [31]
Q38	synthesis	"As no meta-analysis was done, the results from across studies should be described in terms of being similar or dissimilar."	item retrieved from text; no original item; description of item 6: PHAC [31]
Q39	synthesis	"For meta-analyses: Assessment of magnitude and precision of treatment effect"	item 7: PHAC [31]
Q40	outcomes	"Were all of the important outcomes considered? Sufficient information should be provided about adverse outcomes…"	item 9: PHAC [31]
Q41	data integrity	"Were sufficient study characteristics available for both review authors and readers to be able to interpret the results?"	item 3.2 ROBIS [39]
Q42	data integrity	"Were all relevant study results collected for use in the synthesis?"	item 3.3 ROBIS [39]
Q43	general methods	"Were all pre-defined analyses reported or departures explained?"	item 4.2: ROBIS [39]
Q44	outcomes	"Were all the important outcomes considered?"	item 9: SURE [36]
Q45	up-to-date	"Date of review – is it likely to be out of date?"	item 11.2: SURE [36]
Q46	discussion of review findings/results	"Are the conclusions the same in the abstract and the full text?"	item 11.3: SURE [36]

Table 4 Short-list of all items retrieved

This list was compiled after first duplicates had been removed. Duplicates were defined as items with similar wording addressing the same problem in comparison to items already incorporated into healthevidence.org CAT (see Table 3). Items were later reviewed by the authors for eligibility (i.e. new item, content of item used for additional user guidance, or exclusion) and informed the final list of included criteria of CAT HPPR. Not only the question, but also the user guidance of included CATs informed the final CAT HPPR manual.
